# Neutral Theory Predicts the Relative Abundance and Diversity of Genetic Elements in a Broad Array of Eukaryotic Genomes

**DOI:** 10.1371/journal.pone.0063915

**Published:** 2013-06-14

**Authors:** François Serra, Verónica Becher, Hernán Dopazo

**Affiliations:** 1 Evolutionary Genomics Laboratory, Bioinformatics and Genomics Department, Centro de Investigación Príncipe Felipe, Valencia, Spain; 2 Departamento de Computación, Facultad de Ciencias Exactas y Naturales, Universidad de Buenos Aires, Ciudad Universitaria, Buenos Aires, Argentina; 3 Instituto de Genómica Humana—Banco Nacional de Datos Genéticos, Buenos Aires, Argentina; 4 Departamento de Ecología, Genética y Evolución, Facultad de Ciencias Exactas y Naturales, Universidad de Buenos Aires, Ciudad Universitaria, Buenos Aires, Argentina; Universitat Pompeu Fabra, Spain

## Abstract

It is universally true in ecological communities, terrestrial or aquatic, temperate or tropical, that some species are very abundant, others are moderately common, and the majority are rare. Likewise, eukaryotic genomes also contain classes or “species” of genetic elements that vary greatly in abundance: DNA transposons, retrotransposons, satellite sequences, simple repeats and their less abundant functional sequences such as RNA or genes. Are the patterns of relative species abundance and diversity similar among ecological communities and genomes? Previous dynamical models of genomic diversity have focused on the selective forces shaping the abundance and diversity of transposable elements (TEs). However, ideally, models of genome dynamics should consider not only TEs, but also the diversity of all genetic classes or “species” populating eukaryotic genomes. Here, in an analysis of the diversity and abundance of genetic elements in >500 eukaryotic chromosomes, we show that the patterns are consistent with a neutral hypothesis of genome assembly in virtually all chromosomes tested. The distributions of relative abundance of genetic elements are quite precisely predicted by the dynamics of an ecological model for which the principle of functional equivalence is the main assumption. We hypothesize that at large temporal scales an overarching neutral or nearly neutral process governs the evolution of abundance and diversity of genetic elements in eukaryotic genomes.

## Introduction

Species are unevenly represented in ecosystems. In no environment whether terrestrial or aquatic, temperate or tropical, all species are equally common [1]. Species diversity and their relative abundance have always intrigued ecologists [2]. Ecological models of species abundance are basically of two kinds: descriptive (statistical-based) or mechanistic (niche-based or neutral). While many mechanistic approaches assume niche differences as the main cause driving community composition, neutral models consider niche differences among species irrelevant. The unified neutral theory of biodiversity (UNTB) [3], [4], originally inspired by neutral population genetics [5], [6], assumes interactions among trophically similar species as equivalent on an individual or “per capita” basis. This provocative assumption means that the fate of individuals, regardless of the species, appear to be controlled by similar birth, death, dispersal, and speciation rates. Because each species follows a random walk, biodiversity and patterns of relative species abundance emerge by a process of drift in the community. The fundamental biodiversity number 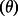, analogous to *4Nμ* of population genetics, governs species richness at large spatial and temporal scales. UNTB is thus a useful null model against to test alternative biological hypotheses for the origin and maintenance of relative species abundance distributions [7], [8].

One can draw an analogy between the distribution of relative species abundance in ecological communities and the distribution of relative abundance of genetic sequences, unique or repetitive, that populate eukaryotic genomes: long terminal repeat (LTR) retrotransposons, non-LTR retrotransposons, cut-and-paste DNA transposons, rolling-circle DNA transposons, self-synthesizing DNA transposons, satellites, simple repeats, tRNA, miRNA, snoRNA and genes among others. For simplicity we will refer to these sequence classes as “genetic species”, the genomic analogue of biological species in community ecology [9]. Dynamical models of genomic diversity have focused on the selective forces shaping the abundance and diversity of transposable elements (TEs) [9]–[11]. Moreover, ecological models of genomes have already been formalized [12], [13], and complex models invoking the genomic equivalent of parasitism, competition and cooperation between TEs [14] have been simulated. However ideally, models of genome dynamics should consider not only TEs, but also the diversity of all genetic species populating eukaryotic genomes. Such model has never been formalized, just as genetic species abundance and diversity has never been predicted under neutrality in genomics.

Does a neutral model with applications in ecology fit the pattern of species abundance and diversity in eukaryotic genomes? How well does such a model perform over the great diversity of eukaryotic genome sizes? To what extent are the abundance and diversity of genome components the result of adaptive or stochastic processes? Here we test the statistical fit of the UNTB predictions in 31 genomes ranging from unicellular eukaryotic species to mammals (Table S1). We organize the results into four sections. First we analyze the relative species abundance (RSA) curves of genomes and chromosomes. Second we simulate the random distribution of genetic elements in chromosomes to test the role of chance in chromosome organization. Third, we test the statistical fit of the UNTB to the relative abundance and diversity of genome elements in chromosomes. Finally, assuming that the chromosome length is the genomic analogue of area in ecology, we ask if the universal ecological species-area relationship is also observed in genomes.

## Results and Discussion

### RSA curves in genomes and chromosomes

Ecologists frequently use RSA curves to compare the richness, the degree of dominance, and the number of rare species in communities. An interesting property of RSA curves is that species are unlabeled so that the RSA distributions of different ecosystems can be compared whatever species they contain. We took advantage of current automatic methods of genome annotation and sequence recognition to build RSA curves for chromosomes (Table S2 and External Dataset 1 at EDS). Genetic species were defined according to biotypes [15], [16], and the “classes” of the RepBase [17] nomenclature.

**Figure 1:**
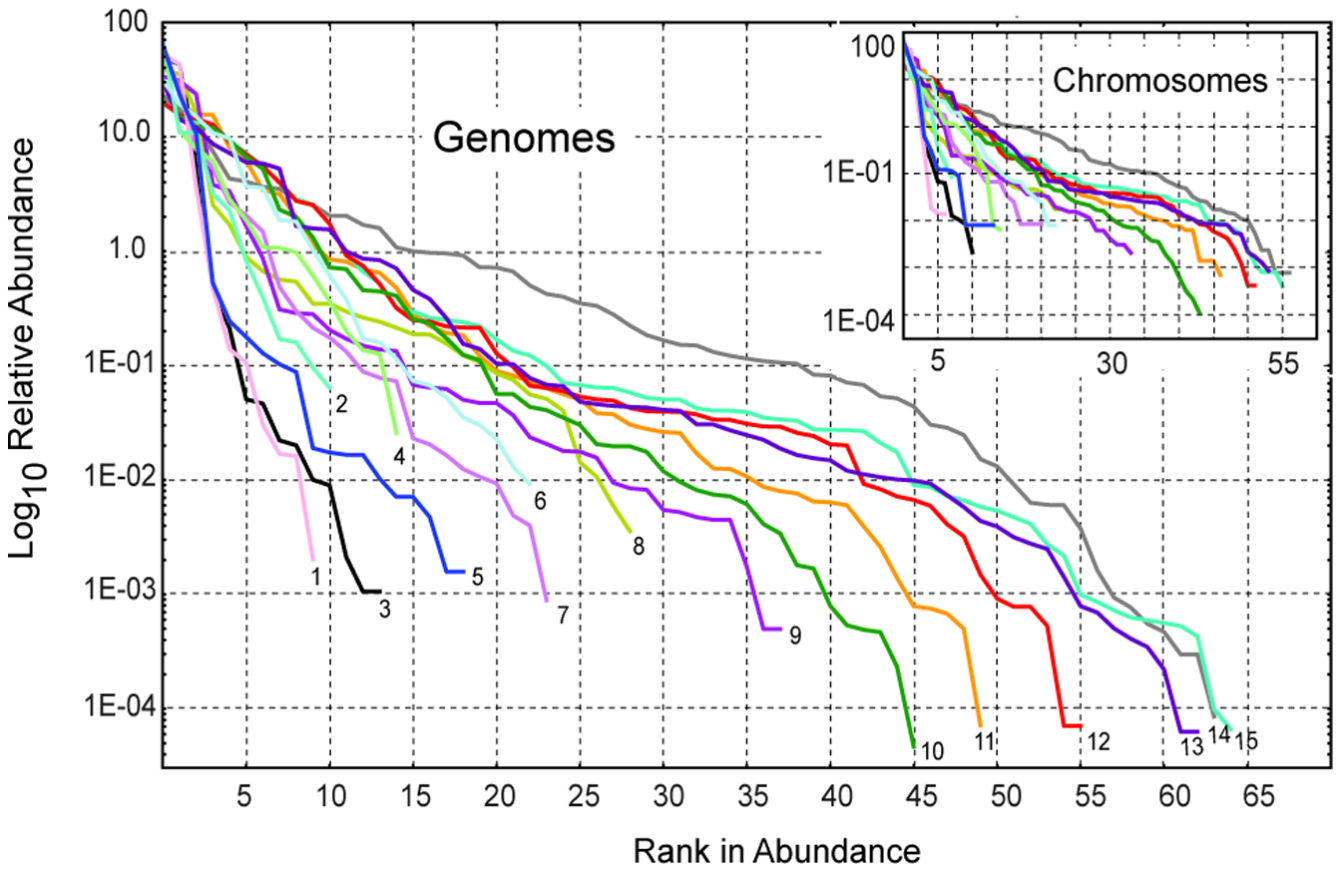
RSA curves in genomes and chromosomes. The figure displays RSA curves for a set of genomes and their largest chromosomes. Note the lower number of genetic species in chromosomes. Genomes and chromosomes are shown in the same color. Numbers correspond to: *1- P. falciparum, 2- S. cerevisae, 3-D. discoideum, 4- P. trichocarpa, 5- T. nigroviridis, 6- S. bicolor, 7- O. latipes, 8- D. melanogaster, 9- G. gallus, 10- M. domestica, 11- C. familiaris, 12- M. mulatta, 13- M. musculus, 14- D. rerio, and 15- H. sapiens*.

Figure 1 displays RSA curves for a subset of genomes and their largest chromosomes (Figure S1 shows the complete set of RSA curves). Although the curves differ in many ways, two patterns are evident: (1) RSA curves of genomes and chromosomes are very similar for each eukaryotic species, and (2) all RSA curves display the universal S-shape observed in ecology [2], [3], where few species are dominating, many are common, while the majority are rare. Both observations suggest a common mechanism of distribution of genetic elements in genomes and chromosomes.

**Figure 2:**
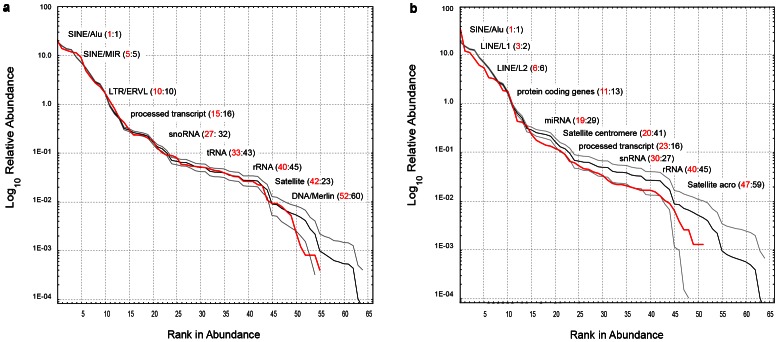
Observed and expected RSA curves. Shifts in the rank of abundance lead to similar distributions of observed and expected RSA curves in chromosomes. Observed abundance (red line), and genome-random distribution (black line, taken from from 1,000 random simulations) of genetic species show a remarkable agreement when plotting RSA curves. Grey lines show two standard deviations corresponding to the variation of the simulated data (black curve). This agreement was observed in almost 80% of the chromosome analyzed, and is mainly due to the frequent shifts in the ranking of abundances of genetic species in chromosomes. The numbers in parenthesis depict the observed (red) and the mean genome-random expected value (black) of some genetic species in chromosomes. Differences between numbers point out over and under abundances of species elements in chromosomes. **a.** Human chromosome 1 (*KS-test*, *P* = 0.51, *n* = 56) **b.** Human chromosome 19 (*KS-test*, *P* = 0.32, *n* = 52).

To what extent does a chromosome's subset of genetic elements represent a random sample of the complete set of elements of the genome? To answer this question, we simulated the random distribution of the full set of elements of a genome among the chromosomes. Based on 1,000 simulation runs, we created expected abundances and standard deviations for each genetic species in each chromosome according to its frequency in the genome. Statistical tests (*t-test*, *FDR*<0.05) established that less than 4% of all genetic species showed abundances in chromosomes that agreed with their random expected distribution (External Dataset S2 at EDS). Therefore, a homogeneous random process cannot account for the observed abundances of genetic species in chromosomes, a result just pointed out for TE's in *D. melanogaster* [9], [10]. However, if the observed RSA curve of each chromosome is superimposed on the mean distribution of 1,000 simulated RSA curves taken from all elements of the genome, a notable agreement is detected. Figure 2 shows this fit for two chromosomes (see External Dataset S3 at EDS for complete results). This remarkable concurrence arises because “genetic species” are unlabeled in the RSA plots, and hence allows compensation by shifts in the ranking order of abundances. Statistical differences between RSA curves were detected for 76 out of 548 chromosomes tested (*KS-test*, *P*<0.05; see External Dataset S2 at EDS2). What is the dynamical mechanism responsible for the 86% agreement of chromosomes tested? Could a neutral dynamical model predict this shared demographic pattern of genomes?

### Testing Hubbell's Neutral Theory in Genomes

Stochastic models are not restricted to physics [18]. Analogous to the kinetic theory of ideal gases, Hubbell's neutral theory of biodiversity is a stochastic theory assuming equivalence among interacting individuals. The theory assumes that diversity in a local community of individuals is in steady state, maintained by a balance between local extinction and immigration from the metacommunity at a constant stochastic rate (*m*). Births and deaths in the local community occur at constant per capita rates regardless of the species. This community reaches a dynamic equilibrium between stochastic extinction and the immigration of new species, while in the metacommunity diversity is maintained by speciation at a single constant rate 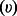 [4], [8].

**Figure 3:**
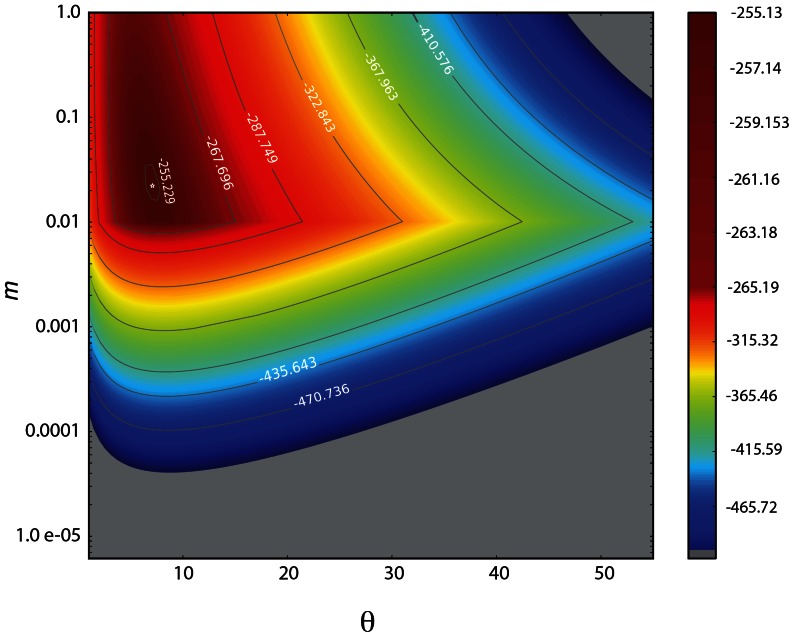
MLE of neutral parameters. Log likelihood surface as a function of migration rate (*m*), and the fundamental biodiversity number 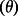 for *Danio rerio* chromosome 19. Dark red shows regions of the surface where parameters maximize the probability to explain abundances and diversity of genetic elements in the chromosome. Likelihood-ratio test favored Etienne's model (in contrast to Ewens' model) as a best explanation of abundance and diversity of genetic elements in this particular chromosome. The dot inside the dark red surface shows coincidence between the analytical and the graphical ML estimation.

For genomes, we assume that each chromosome is the physical arena in which genetic elements spread, degenerate and are replaced by other elements of the same or different species. Genetic elements could come from the same chromosome, or from any other chromosome of the genome. As a first approximation, we assume that each chromosome represents a local community of *J* elements and *S* different genetic species while the rest of chromosomes correspond to the metacommunity of size *J_M_*. Given the total number of genetic elements in each chromosome we optimized the neutral parameters *m* and θ ( = 2*J_M_* ν) of Ewens [19] and Etienne's [20] sampling formulas using maximum-likelihood estimation (Figure 3). These models were optimized using *EcoloPy-UNTBgen* program, a new software based in previous programs [20]–[22], and especially designed to test for neutrality with large genomic datasets (see methods). Since Ewens' model is a special case of the Etiennes' model, we compared the parameter of likelihood-ratio test (LRT) [23] to choose the best-fit model to the data. With best-fitted parameters we simulated 10,000 neutral distributions of genetic elements for each chromosome and test for neutrality.

**Figure 4:**
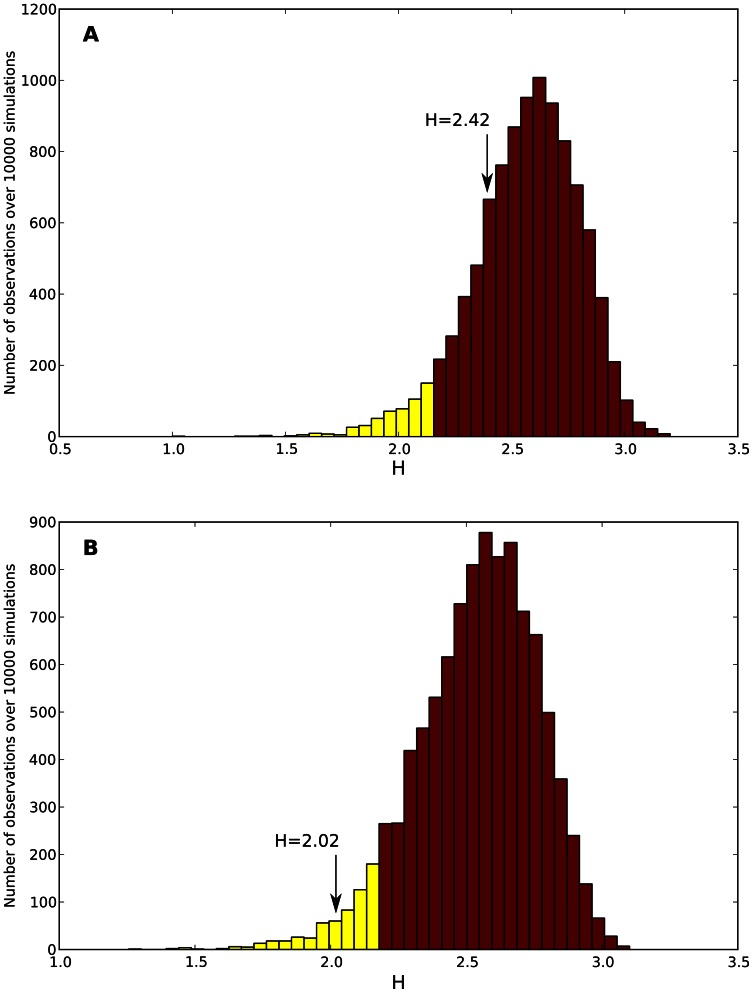
Comparing simulated and empirical evenness. Neutrality test compares null distributions of H against the empirical H value of the corresponding chromosome. In this case, the null distribution of H values was derived from 10,000 neutral simulations of (A) *Homo sapiens* chromosome 1 and (B) *Anopheles gambiae* chromosome 2L, with neutral parameters (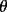 and *m*) estimated by ML optimization using the Etienne's sampling formula. Yellow and red bars display 5% and 95% of the simulated neutral data, respectively. Although neutrality was not rejected in B (*p* = 0.291 and *p* = 0.041 for A and B respectively), posterior correction by multiple testing favored the neutral hypothesis (*q* = 0.609 and *q* = 0.159 for A and B respectively). Only 31 out of 548 (5.6%) chromosomes showed significant deviations previous FDR correction.

To test the UNTB, we computed the evenness (*H*) of each simulated distribution according to the methodology of Jabot and Chave [21] (see methods). We confronted the set of *H* values with the simulated distribution to assess whether the empirical *H* values of chromosomes lie outside the confidence limits of the neutral expectation (Figure 4). We considered chromosomes to be significantly neutral if the empirical evenness of the chromosome was within the 95% of the 10,000 random neutral values. We observed deviations from neutrality in 31 out of 548 chromosomes (5.6%) However, significant deviations vanished after correction for multiple testing (*FDR*<0.05, *n* = 548) [24]. This result was robust even when genetic species were defined at different levels of the hierarchy of the RepBase ontology, and even statistically, when an alternative test of neutrality was implemented [25] (see methods).

Table 1 displays a representative sample of the results (for all results see External Dataset S4 at EDS). According to these results, Hubbell's neutral model is a sufficient model to explain the abundance and diversity of genetic elements in each chromosome of all the 31 eukaryotic genomes we analyzed.

**Table 1:** Neutral parameters estimation.

Species	Ch	J	S	ΔH	θ	*m*	P (Q) -val	Best-model
*Tribolium castaneum*	7	7,865	18	−0.97	2.12	-----	0.01 (0.66)	Ewens
*Anopheles gambiae*	X	21,215	42	−0.58	6.97	0.037	0.03 (0.66)	Etienne
*Gallus gallus*	9	6,621	32	−0.56	4.28	-----	0.05 (0.66)	Ewens
*Drosophila melanogaster*	X	20,787	26	−0.51	2.86	-----	0.09 (0.66)	Ewens
*Tetraodon nigroviridis*	3	8,505	17	−0.49	1.96	-----	0.11 (0.66)	Ewens
*Mus musculus*	14	143,018	59	−0.27	6.58	0.149	0.15 (0.66)	Etienne
*Populus trichocarpa*	2	32,946	15	−0.37	2.23	0.009	0.16 (0.66)	Etienne
*Oryzias latipes*	19	7,223	21	−0.35	2.57	-----	0.17 (0.66)	Ewens
*Homo sapiens*	17	93,105	52	−0.24	6.51	0.065	0.18 (0.66)	Etienne
*Macaca mulatta*	16	84,626	50	−0.19	5.95	0.119	0.23 (0.66)	Etienne
*Saccharomyces cerevisiae*	II	640	9	−0.25	1.37	-----	0.26 (0.66)	Ewens
*Dictyostelium discoideum*	1	26,650	14	−0.24	1.36	-----	0.27 (0.66)	Ewens
*Danio rerio*	1	105,305	56	−0.11	8.17	0.016	0.29 (0.66)	Etienne
*Canis familiaris*	1	144,103	47	−0.10	5.28	0.093	0.32 (0.66)	Etienne
*Plasmodium falciparum*	13	18,738	10	−0.11	0.95	-----	0.38 (0.66)	Ewens
*Monodelphis domestica*	2	675,788	44	−0.02	4.46	0.031	0.43 (0.66)	Etienne
*Sorghum bicolor*	1	37,626	23	0.19	2.86	0.067	0.68 (0.79)	Etienne

The table depicts parameters and statistics estimated for a selection of chromosomes of different species. Chromosomes are arranged according to *p*-value from the less to the most neutral. J is the total number of genetic elements; S is the number of genetic species; ΔH is the difference between the observed and the expected evenness (Shannon's diversity index); θ is the fundamental diversity number; *m* is the migration rate; *P* and *Q*-val are statistical significances of the neutral test before and after false-discovery rate correction. The last column shows the model (Ewens or Etienne) that best fitted the empirical distribution of genetic elements in the chromosome after likelihood-ratio test (*p*<0.05, *df* = 1). After multiple-testing correction (*FDR*<0.05), none of the 548 chromosomes of the 31 eukaryotic genomes showed significant deviations from neutrality.

To assess the power of this conclusion we simulated neutral and log-normal distributions as null and alternative hypotheses along a wide range of species (*S*) and individuals (*J*). The proportion of times the test failed to reject the null hypothesis being false was 50% for small chromosomes and communities (*J<25,000*, and *S<30*, Figure S2A). Conversely, the percentage of times the test failed to accept the null hypothesis being true was low (<5%) for a wide range of *S* and *J* values (Figure S2B). Therefore, for large neutral communities the neutral test employed provided a robust positive answer with high power.

### Genome species-area relationship

**Figure 5:**
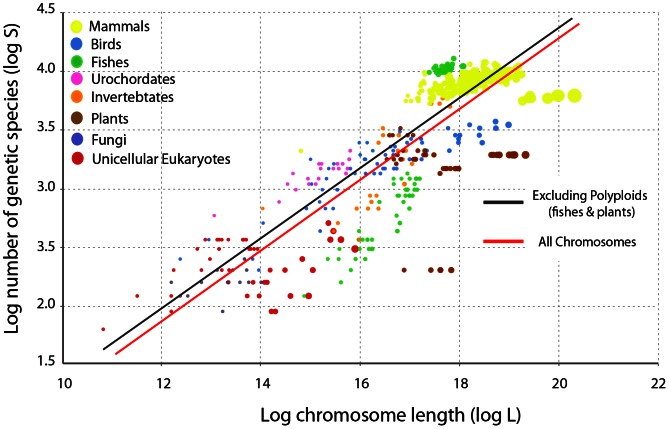
Genetic species and chromosome length. Analogous to ecological communities, eukaryotic chromosomes display the universal relationship between species number and area observed in ecosystems. As expected, when polyploid species are excluded from the analysis the adjustment to the model is better (see text). Excluding polyploid species (*n* = 412), log(S) = −1.97+0.31 log(L), *R^2^* = 0.87, *P*<<0.01. Including all chromosomes (*n* = 548), log(S) = −2.07+0.32 log(L), *R^2^* = 0.73, *P*<<0.01. Note that for simplicity, the set of polyploid species considered were the pull of fish and plants. A significant outlier is *Danio rerio* that stands upwards the correlation line, with the highest number of genetic species observed in fishes.

If a purely neutral stochastic process controls the abundance and diversity of genetic elements in chromosomes, it is expected that S, the number of genetic species, will increase with chromosome length. In ecology it is universally observed that larger areas contain more species. Does this pattern hold true for genomes and chromosomes? The standard species-area relationship in ecology is the Arrhenius power law [26] *S* = *c*A*^z^*, where *S* is the species number, A is the area and *c* and *z* are constants. Considering a fixed length of chromosome, others things being equal, it is expected that non-polyploid species show a higher number of genetic species than polyploids, and therefore a better statistical fit to the species-area relationship. After least square fit of the power function we effectively observed *c* = 0.28, *z* = 0.27 (*R^2^* = 0.64 *n* = 548) for all chromosomes studied including polyploids (figured, for simplicity, by fish and plant species), and *c* = 0.50, *z* = 0.25 (*R^2^* = 0.81, *n* = 412) excluding them. For relatively recent polyploid species, there was no sufficient time of divergence to produce genetic species according to the neutral expectations. Figure 5 displays the log-log transformation of both curves. In both cases, the adjustment was statistically significant by a linear regression model (Pearson, *P*<<0.001). As in community ecology, eukaryotic chromosomes display the universal species-area relationship with *z* values analogous to regional spatial scales [27].

## Conclusion

Almost one hundred years ago ecologists recognized the universal uneven distribution of species abundance, and the increase in number of species with increasing area [1]. Just a decade ago, however, neutral demographic processes emerged as the simplest mechanical explanation behind both patterns in communities [3]. More recently, Lynch and Conery [28] hypothesized that complexity of eukaryotic genomes emerged passively during evolution as a consequence of population size reduction. Here, we demonstrated that a simple stochastic process associated to a few number of parameters fits the pattern of abundance and diversity of genetic species along a great diversity of eukaryotic genomes.

We are certainly aware that the fit of a neutral pattern does not necessarily imply the existence of a neutral process behind the pattern, but it does offer the simplest explanation consistent with current data. The excellent, taxonomically broad fit of neutral theory to genomic element diversity and abundance raises the unavoidable question: why is there not a stronger signature of natural selection in ecological communities or in genomes at large scales? Ecologists have recognized the existence of many kinds of trade-offs, for instance species with high dispersal rates are not good competitors. However, it is not yet known to what extent such trade-offs maintain diversity or is consistent with, neutral dynamics. For genomes, the mechanisms that maintain element diversity, and whether these involve trade-offs, are not yet understood. Which mechanisms operate will also depend on whether genome size is under strong or weak selection [29]. More likely, element diversity of genomes results from some combination of neutral drift and weak selection on different genetic species [30]. Independently of the answer, the model tested here should be the null hypothesis to test for alternative mechanisms explaining the community dynamics of genetic elements in eukaryotic genomes.

## Methods

### Genomes, Genetic Species and Elements

Genome sequences of 31 species ranging from unicellular eukaryotes to mammals were downloaded from Ensembl [16] (Table S1). Genetic elements of genomes were divided in biotypes and repeated elements. Repeated elements in chromosomes were retrieved using RepeatMasker program (http://www.repeatmasker.org) with default parameters. Biotypes were retrieved using the Biomart API [15], and each type was used as genetic species (GS). Pseudogenes were removed from the analysis. For repetitive elements, the “class” level of the hierarchy of the RepBase ontology [17], also referred to as “superfamily”, according to the International Committee on Classification of Transposable Elements (http://giri.org) was considered here a “genetic species” (GS). Each of the elements of a GS (repetitive element or biotype) was considered a genetic element of a genome.

Table S2 summarizes the information of species and number of elements for two chromosomes. The complete set of genetic species and the corresponding number of elements for each chromosome in all genomes is available in External Dataset S1 at EDS.

### Randomization of genetic elements in chromosomes

To test for the random allocation of genetic elements in chromosomes we generated 1,000 random distributions of genetic elements in each chromosome for all genomes. Simulations considered chromosome size as the sum of all 10 kb windows containing at least 1 genetic element; although this methodology is simple, it ensures to avoid wired regions as centromeres or not fully sequenced regions. All genetic elements of each genome were distributed among chromosomes, according to a probability dependent of the size of the chromosome. Therefore, for genetic elements of the human genome there is a six times greater likelihood of belonging to chromosome 1 than to chromosome 22 (since their respective lengths are 225 Mb and 35 Mb).

### EcoloPy-UNTBgen Package

There are specific tools for the statistical analysis of species abundances data in ecology. Many of them implement statistical functions fitting data to models and testing for neutrality [21], [22]. However none of these programs are able to deal with the large dataset characteristic of genomes. The high number of repetitive elements in genomes makes impractical most algorithms, especially the computation of the Etienne model described below. In order to adapt the algorithm to large samples, we developed *UNTBgen* in *EcoloPy* package (http://bioinfo.cipf.es/ecolopy/). *UNTBgen* implements all functions of UNTB [22] and the Etiennes's programs [20] which were written in R language and in PARI/GP system, respectively. However, *UNTBgen* takes advantage of the GNU Multiple Precision (GMP) library [31], and the Multiple-Precision Binary Floating-Point (MPFR) library [32], through the fast multiprecision GMPY module.

### Ewens and Etienne models

Ewens sampling formula (ESF) [19] describes the probability distribution of a configuration of alleles in a sample of genes under the infinite-alleles model. By defining 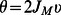 (3) it is possible to apply ESF and to compute its likelihood given a community of individual of different number of species,
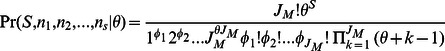
where, *S* is the total number of species, *n_i_* corresponds to the abundance of species *i* and *φ_a_* the number of species with abundance *a*. *UNTBgen* is able to generate, using this sampling formula a neutral distribution of species abundances given sample size *J* and *θ*. The number of species generated is free according to the formula but can be fixed by keeping only those random abundances generated with the desired number of species. The likelihood function:
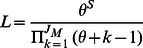
is also integrated in *UNTBgen* program, and used for optimization of the *θ* parameter. Hubbell's analytical results of ESF considered unlimited dispersal of individuals (*m* = 1). Subsequently, Etienne established a new sampling formula with limited dispersal (*m*<1), thus:
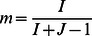
where *I* is the number of immigrants that compete with the local individuals for vacant spots in a fixed area size after the death of individual in the local community.

Etienne's sampling formula is:
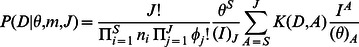
where,
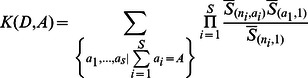
where 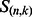 is the stirling number of the generic *n* and *k* values. This computation is the main computation bottleneck in the resolution of the equation, as was mentioned by Etienne [20]. *UNTBgen* performs the same calculation implemented by Jabot and Chave in the program Tetame [33] that takes advantage of the recurrence function of stirling numbers:
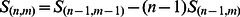
This function allows building a table of stirling numbers instead of computing them directly (in order to compensate the memory requirements of genomic data, this table may be dynamically reduced to the needed values of *n* and *k*). After computation, *UNTBgen* computes the likelihood of Etienne's sampling formula according to the parameters *θ* and *m* for a given dataset allowing parameters optimization.

### Model optimization and testing

Ewens and Etienne models were optimized through different optimization strategies. In the case of the Ewens' formula, *θ* is the only parameter to be considered, and its estimation was achieved with the golden optimization strategy [34]. For Etienne's model, two parameters were optimized, *θ* and *m*. In this case we used different strategies implemented in Scipy [31], the best solution among the downhill simplex algorithm [35], the L-BFGS-B algorithm [36], the truncated Newton algorithm and the Sequential Least Squares Programming.

Parameter optimization is a critical step especially under Etienne's model. Under this model the optimization of the parameters may lead to several optima [37]; a simple way to check if our estimation of the global optima is true consists in exploring the likelihood surface representative of the variation of the parameters studied. We thus followed this procedure given a range of values of *θ* and *m* for representative chromosomes in our dataset. The inspection of the likelihood surface generated (Figure S2) allows us to find the best solution for both parameters. Since Ewens' model is a special case of Etienne's model, we compared the delta parameter of the likelihood ratio test (LRT) against the value of a chi-squared distribution with one degree of freedom. Additionally, given the large number of statistical tests performed, we applied a Bonferoni correction to the number of results showing statistical significance to correct for Type I error (FDR<0.05) [38]. Etienne's model was thus kept as best fit model for only those chromosomes that pass the LRT after FDR adjustment; otherwise the null model using Ewens' formula was selected.

### Test of neutrality

Recently two exact tests were developed to accept or reject the neutral community ecology hypothesis [21], [25]. Both tests are based on the simulation of a given number of random neutral communities, with parameters estimated from the actual data, and the posterior comparison with the observed distribution of abundance.

The first test [25] compares likelihood distributions. This corresponding distribution of random neutral abundances is compared to the likelihood of the observed data. The major problem of this test is technical. The machine computation time needed to calculate the K(D,A) from thousands of neutral simulations with genomic data make this approximation impractical. However, in order to ensure the robustness of our results, we applied this test using 100 simulations (Table S3).

The second test [21] compares, instead of likelihood, the Shannon's entropy [39] index (sometimes called evenness):
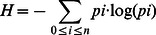
where, *n* is the total number of genetic species in chromosome and *pi* is the fraction of individuals belonging to the species *i* in that chromosome. This approach is faster because random abundances do not need to be optimized in the neutral model.

## Supporting Information

Figure S1 **The full set of 31 RSA curves for all genomes analyzed.**
(TIFF)

Figure S2 **Neutral Test Validation.** False positive and false negative results of the neutral test were assessed for a variable range of species (*S*) and individuals (*J*). Neutral and log-normal distributions were assumed as null and alternative hypotheses, respectively. Panel A describes the proportion of times the test rejected the null hypothesis being true. Red regions describe the space where the proportion of false positive is too high. This is a dangerous area to test for neutrality. Panel B shows the percentage of times the test failed to accept the null hypothesis being true. Intersection between horizontal and vertical lines correspond to the results of 200 simulations with the corresponding J and S. Data of chromosomes and ecological communities are pointed in both panels: (1) *A. thaliana* chr1, (2) *D. rerio* chr1, (3) *D. discoideum* chr2, (4) *C. elegans* chrI, (5) *D. melanogaster* chr2L, (6) *G. gallus* chr18, (7) *G. gallus* chr2, (8) *H. sapiens* chr1, (9) *H. sapiens* chr21, (10) *Z. mays* chr1, (11) *Z. mays* chr3, (12) *M. musculus* chr10, (13) *M. domesticus* chr1, (14) *M. domesticus* chr3, (15) *M. domesticus* chr5, (16) *P. falciparum* chr13, (17) *R. norvegicus* chr1, (18) *S. bicolor* chr7, (19) *T. nigroviridis* chr9, (20) *T. castaneum* chr8, (21) BCI, (22) Edoro, (23) La Planada, (24) Lambir, (25) Lenda, (26) Mudamalai, (27) Pasoh, (28) Sinharaja and (29) Yasuni.
(TIF)

Table S1 **Eukaryotic genomes and databases.** Features: AP: Ancient Polyploid; LGS: Largest Genome Sequenced; RG: Reduced genome RP: Recent Polyploid; UE: Unicellular Eukaryote. -1-: http://www.hgsc.bcm.tmc.edu/ftp-archive/Tcastaneum/Tcas3.0/.
(DOCX)

Table S2 **Genetic species and number of elements in two selected chromosomes.** Genetic species are arranged according to the observed ranking of abundance.
(DOCX)

Table S3 **Optimization of Models and Parameters.** The columns “theta Etienne” and “theta Ewens” show the values of θ calculated according to Etienne model Ewens sampling formula, respectively. The “m” column shows the migration parameter according to Etienne's model. “Best model” column shows which model was found to have a better fit for each chromosome. “lnL p(q)-val” column corresponds to the p-value (q-value) of the neutrality test comparing the likelihood to fit Etienne's model of simulated data to the likelihood of observed data. “N” column shows the number of simulations that where generated for each chromosome (in the context of the neutrality test comparing likelihoods). “delta-H” represents the difference in shannon entropy (H) between 1,000 simulation and the observed values. “shannon p(q)-val” column shows the p-value(q-value) of the neutrality test that consists in comparing the shannon entropy of simulated distribution of species' abundances to the observed data.
(DOCX)

## References

[1] Magurran A (2004) Measuring biological diversity. Oxford, UK: Blackwell Science Ltd.

[2] McGill BJ, Etienne RS, Gray JS, Alonso D, Anderson MJ, et al (2007) Species abundance distributions: moving beyond single prediction theories to integration within an ecological framework. Ecology Letters 10: 995–1015 doi:10.1111/j.1461-0248.2007.01094.x 17845298 10.1111/j.1461-0248.2007.01094.x

[3] Hubbell SP (2001) The unified neutral theory of biodiversity and biogeography. Princeton Univ Dept of Art & 1. 10.1016/j.tree.2011.03.024 21561679

[4] Rosindell J, Hubbell SP, Etienne RS (2011) The unified neutral theory of biodiversity and biogeography at age ten. Trends Ecol Evol (Amst) 26: 340–348 doi:10.1016/j.tree.2011.03.024 21561679 10.1016/j.tree.2011.03.024

[5] Kimura M (1985) The Neutral Theory of Molecular Evolution. Cambridge Univ Pr

[6] Wright S (1931) Evolution in Mendelian Populations. Genetics 16: 97. 17246615 10.1093/genetics/16.2.97 PMC1201091

[7] Volkov I, Banavar JR, Hubbell SP, Maritan A (2003) Neutral theory and relative species abundance in ecology. Nature 424: 1035–1037 doi:10.1038/nature01883 12944964 10.1038/nature01883

[8] Alonso D, Etienne RS, McKane AJ (2006) The merits of neutral theory. Trends Ecol Evol (Amst) 21: 451–457 doi:10.1016/j.tree.2006.03.019 16766082 10.1016/j.tree.2006.03.019

[9] Bartolomé C, Maside X, Charlesworth B (2002) On the abundance and distribution of transposable elements in the genome of Drosophila melanogaster. Mol Biol Evol 19: 926–937. 12032249 10.1093/oxfordjournals.molbev.a004150

[10] Rizzon C, Marais G, Gouy M, Biémont C (2002) Recombination Rate and the Distribution of Transposable Elements in the Drosophila melanogaster Genome. Genome Res 12: 400–407 doi:10.1101/gr.210802 11875027 10.1101/gr.210802 PMC155295

[11] Le Rouzic A, Boutin TS, Capy P (2007) Long-term evolution of transposable elements. Proc Natl Acad Sci USA 104: 19375–19380 doi:10.1073/pnas.0705238104 18040048 10.1073/pnas.0705238104 PMC2148297

[12] Brookfield JFY (2005) The ecology of the genome - mobile DNA elements and their hosts. Nat Rev Genet 6: 128–136 doi:10.1038/nrg1524 15640810 10.1038/nrg1524

[13] Venner S, Feschotte C, Biémont C (2009) Dynamics of transposable elements: towards a community ecology of the genome. Trends Genet 25: 317–323. 19540613 10.1016/j.tig.2009.05.003 PMC2945704

[14] Leonardo TE, Nuzhdin SV (2002) Intracellular battlegrounds: conflict and cooperation between transposable elements. Genet Res 80: 155–161. 12688654 10.1017/s0016672302009710

[15] Kinsella RJ, Kähäri A, Haider S, Zamora J, Proctor G, et al (2011) Ensembl BioMarts: a hub for data retrieval across taxonomic space. Database (Oxford) 2011: bar030 doi:10.1093/database/bar030 21785142 10.1093/database/bar030 PMC3170168

[16] Flicek P, Amode MR, Barrell D, Beal K, Brent S, et al (2011) Ensembl 2011. Nucleic Acids Res 39: D800–D806 doi:10.1093/nar/gkq1064 21045057 10.1093/nar/gkq1064 PMC3013672

[17] Kapitonov VV, Jurka J (2008) A universal classification of eukaryotic transposable elements implemented in Repbase. Nature Publishing Group 9: 411–2–authorreply414 doi:10.1038/nrg2165-c1 10.1038/nrg2165-c1 18421312

[18] Blythe RA, McKane AJ (2007) Stochastic models of evolution in genetics, ecology and linguistics. J Stat Mech 2007: P07018–P07018 doi:10.1088/1742-5468/2007/07/P07018

[19] Ewens W (1972) The sampling theory of selectively neutral alleles. Theoretical Population Biology 3: 87–112 doi:10.1016/0040-5809(72)90035-4 4667078 10.1016/0040-5809(72)90035-4

[20] Etienne R (2005) A new sampling formula for neutral biodiversity. Ecology Letters 8: 253–260.

[21] Jabot F, Chave J (2011) Analyzing tropical forest tree species abundance distributions using a nonneutral model and through approximate Bayesian inference. The American Naturalist 178: E37–E47 doi:10.1086/660829 10.1086/660829 21750378

[22] Hankin R (2007) Introducing untb, an R Package For Simulating Ecological Drift Under the Unified Neutral Theory of Biodiversity. Journal of Statistical Software 22: 15pp.

[23] Wilks S (1938) The large-sample distribution of the likelihood ratio for testing composite hypotheses. The Annals of Mathematical Statistics 9: 60–62.

[24] Benjamini Y, Hochberg Y (1995) Controlling the false discovery rate: a practical and powerful approach to multiple testing. Journal of the Royal Statistical Society Series B (Methodological) 289–300.

[25] Etienne RS (2007) A neutral sampling formula for multiple samples and an “exact” test of neutrality. Ecology Letters 10: 608–618 doi:10.1111/j.1461-0248.2007.01052.x 17542939 10.1111/j.1461-0248.2007.01052.x

[26] Arrhenius O (1921) Species and Area. Journal of Ecology 9: 95–99.

[27] Rosenzweig ML (1995) Species diversity in space and time. Cambridge. UK: Cambridge University Press.

[28] Lynch M, Conery JS (2003) The origins of genome complexity. Science 302: 1401–1404 doi:10.1126/science.1089370 14631042 10.1126/science.1089370

[29] Cavalier-Smith T (2005) Economy, speed and size matter: evolutionary forces driving nuclear genome miniaturization and expansion. Annals of Botany 95: 147–175 doi:10.1093/aob/mci010 15596464 10.1093/aob/mci010 PMC4246715

[30] Ohta T (2011) Near-neutrality, robustness, and epigenetics. Genome Biol Evol 3: 1034–1038 doi:10.1093/gbe/evr012 21979156 10.1093/gbe/evr012 PMC3227401

[31] Granlund T (2008) The GNU Multiple Precision Arithmetic Library, version 4.2. 2.

[32] Fousse L, Hanrot G, Lefèvre V, Pélissier P, Zimmermann P (2007) MPFR. ACM Trans Math Softw 33: 13–es doi:10.1145/1236463.1236468

[33] Jabot F, Etienne RS, Chave J (2008) Reconciling neutral community models and environmental filtering: theory and an empirical test. Oikos 117: 1308–1320 doi:10.1111/j.2008.0030-1299.16724.x

[34] Jones E, Oliphant T, Peterson P (2001) others (2001) SciPy: Open source scientific tools for Python.

[35] Nelder J (1965) A Simplex Method for Function Minimization. The computer journal

[36] Byrd R, Lu P, Nocedal J (1995) A limited memory algorithm for bound constrained optimization. …. Journal on Scientific Computing

[37] Etienne RS (2006) Comment on “Neutral Ecological Theory Reveals Isolation and Rapid Speciation in a Biodiversity Hot Spot.”. Science 311: 610b–610b doi:10.1126/science.1121914 16456064 10.1126/science.1121914

[38] Benjamini Y, Drai D, Elmer G, Kafkafi N, Golani I (2001) Controlling the false discovery rate in behavior genetics research. Behav Brain Res 125: 279–284. 11682119 10.1016/s0166-4328(01)00297-2

[39] Shannon C (1948) A mathematical theory of communication. Bell System Technical Journal 27: 379–656, 379-423, 623-656.

