# Supplementary figures and images for: Neutral Theory Predicts the Relative Abundance and Diversity of Genetic Elements in a Broad Array of Eukaryotic Genomes
Source: PLoS One. 2013 Jun 14;8(6):e63915. doi: 10.1371/journal.pone.0063915 (PMC3683013; doi:10.1371/journal.pone.0063915)

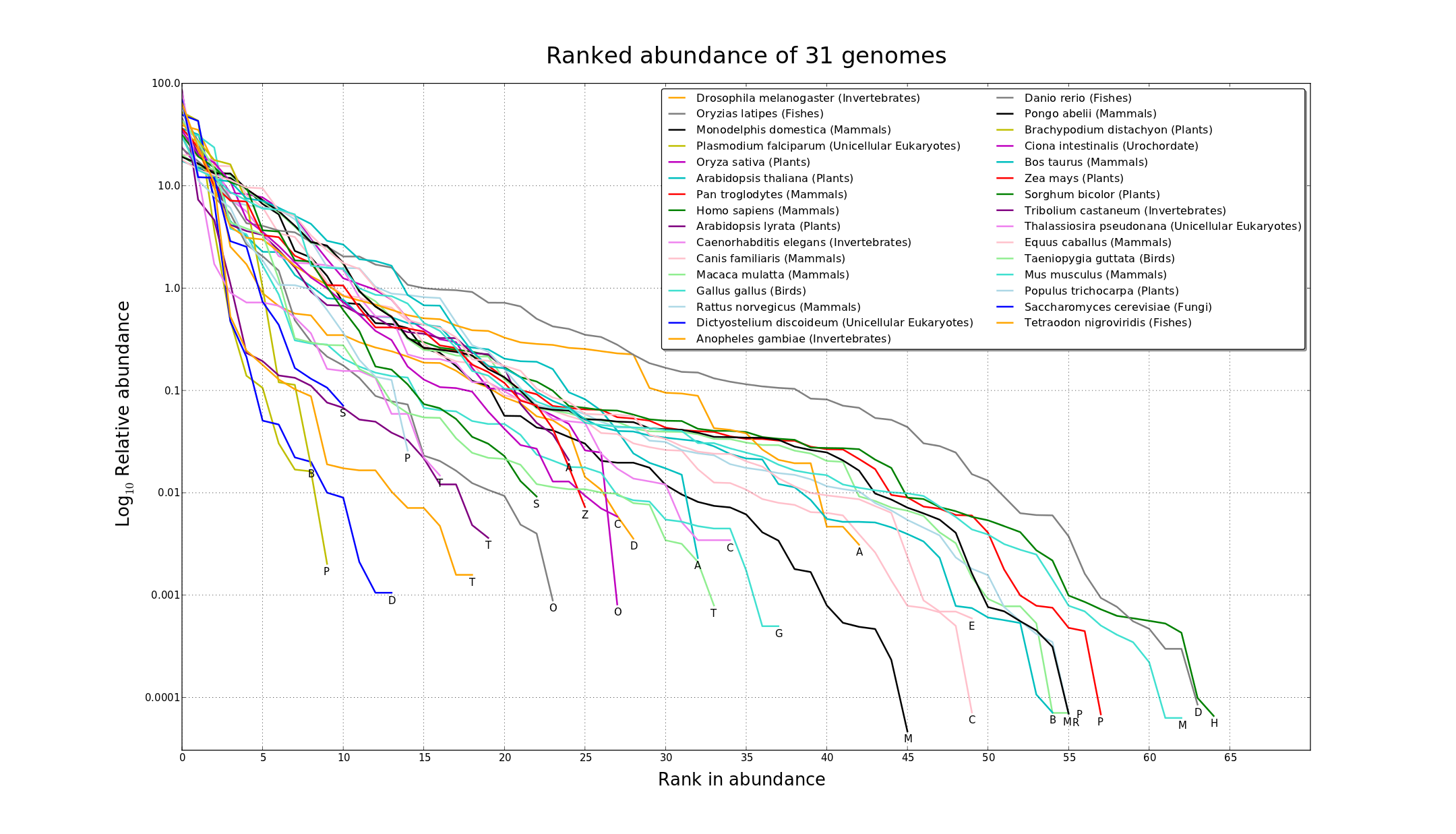

Supplement: Figure S1 — The full set of 31 RSA curves for all genomes analyzed. (TIFF) [file pone.0063915.s001.tiff]

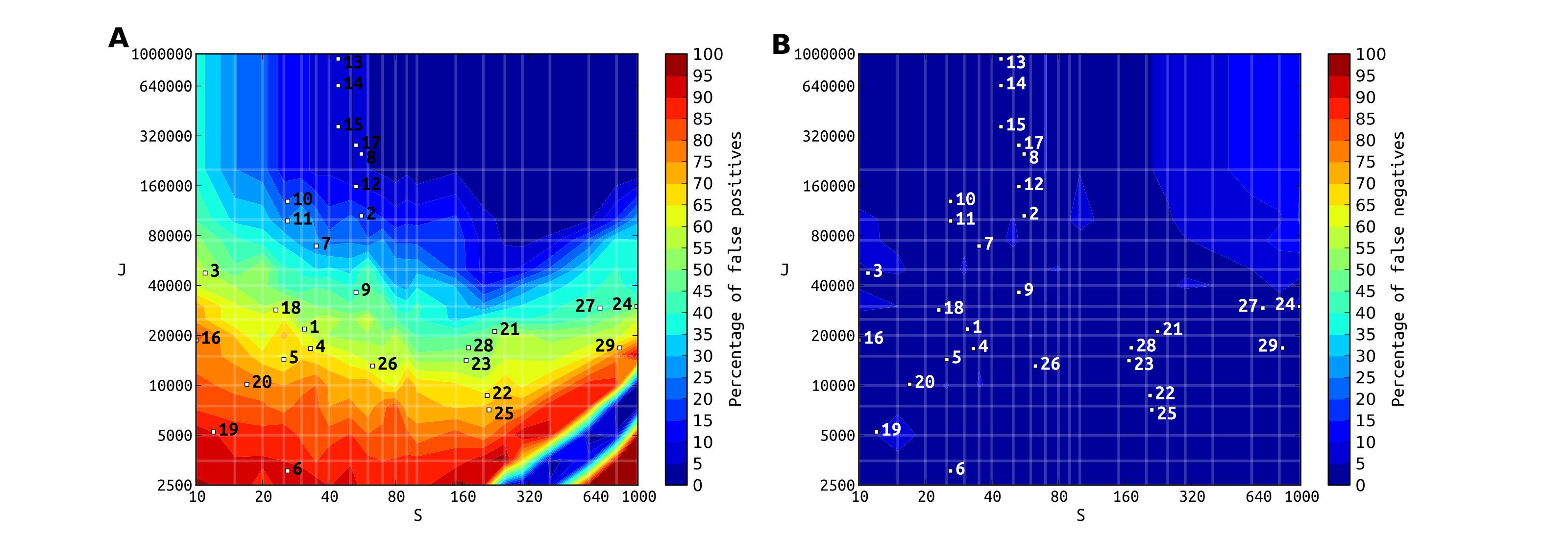

Supplement: Figure S2 — Neutral Test Validation. False positive and false negative results of the neutral test were assessed for a variable range of species (S) and individuals (J). Neutral and log-normal distributions were assumed as null and alternative hypotheses, respectively. Panel A describes the proportion of times the test rejected the null hypothesis being true. Red regions describe the space where the proportion of false positive is too high. This is a dangerous area to test for neutrality. Panel B shows the percentage of times the test failed to accept the null hypothesis being true. Intersection between horizontal and vertical lines correspond to the results of 200 simulations with the corresponding J and S. Data of chromosomes and ecological communities are pointed in both panels: (1) A. thaliana chr1, (2) D. rerio chr1, (3) D. discoideum chr2, (4) C. elegans chrI, (5) D. melanogaster chr2L, (6) G. gallus chr18, (7) G. gallus chr2, (8) H. sapiens chr1, (9) H. sapiens chr21, (10) Z. mays chr1, (11) Z. mays chr3, (12) M. musculus chr10, (13) M. domesticus chr1, (14) M. domesticus chr3, (15) M. domesticus chr5, (16) P. falciparum chr13, (17) R. norvegicus chr1, (18) S. bicolor chr7, (19) T. nigroviridis chr9, (20) T. castaneum chr8, (21) BCI, (22) Edoro, (23) La Planada, (24) Lambir, (25) Lenda, (26) Mudamalai, (27) Pasoh, (28) Sinharaja and (29) Yasuni. (TIF) [file pone.0063915.s002.tif]
