# Supplementary material for: Neutral Theory Predicts the Relative Abundance and Diversity of Genetic Elements in a Broad Array of Eukaryotic Genomes
Source: PLoS One. 2013 Jun 14;8(6):e63915. doi: 10.1371/journal.pone.0063915 (PMC3683013; doi:10.1371/journal.pone.0063915)
Supplement: Table S1 — Eukaryotic genomes and databases. Features: AP: Ancient Polyploid; LGS: Largest Genome Sequenced; RG: Reduced genome RP: Recent Polyploid; UE: Unicellular Eukaryote. -1-: http://www.hgsc.bcm.tmc.edu/ftp-archive/Tcastaneum/Tcas3.0/. (DOCX) [file pone.0063915.s003.docx]

| **Features** | **Species** | **Version** | **Clade** |
| --- | --- | --- | --- |
|  | *Anopheles gambiae* | Ensembl Metazoa 9 | Invertebrates |
| AP | *Arabidopsis lyrata* | Ensembl Plants 9 | Plants |
| AP RG | *Arabidopsis thaliana* | Ensembl Plants 9 | Plants |
|  | *Bos taurus* | Ensembl Vertebrates 62 | Mammals |
| AP | *Brachypodium distachyon* | Ensembl Plants 9 | Plants |
|  | *Caenorhabditis elegans* | Ensembl Metazoa 9 | Invertebrates |
|  | *Canis familiaris* | Ensembl Vertebrates 62 | Mammals |
|  | *Ciona intestinalis* | Ensembl Vertebrates 62 | Urochordate |
| AP | *Danio rerio* | Ensembl Vertebrates 62 | Fishes |
| UE | *Dictyostelium discoideum* | Ensembl Protists 9 | Amebozoa |
|  | *Drosophila melanogaster* | Ensembl Metazoa 9 | Invertebrates |
|  | *Equus caballus* | Ensembl Vertebrates 62 | Mammals |
|  | *Gallus gallus* | Ensembl Vertebrates 62 | Birds |
| hjhj | *Homo sapiens* | Ensembl Vertebrates 62 | Mammals |
|  | *Macaca mulatta* | Ensembl Vertebrates 62 | Mammals |
| LGS | *Monodelphis domestica* | Ensembl Vertebrates 62 | Mammals |
|  | *Mus musculus* | Ensembl Vertebrates 62 | Mammals |
| AP | *Oryza sativa* | Ensembl Plants 9 | Plants |
| AP | *Oryzias latipes* | Ensembl Vertebrates 62 | Fishes |
|  | *Pan troglodytes* | Ensembl Vertebrates 62 | Mammals |
| UE | *Plasmodium falciparum* | Ensembl Protists 9 | Ampicomplexa |
|  | *Pongo abelii* | Ensembl Vertebrates 62 | Mammals |
| AP | *Populus trichocarpa* | Ensembl Plants 9 | Plants |
|  | *Rattus norvegicus* | Ensembl Vertebrates 62 | Mammals |
| AP | *Saccharomyces cerevisiae* | Ensembl Fungi 3 | Fungi |
| AP | *Sorghum bicolor* | Ensembl Plants 9 | Plants |
|  | *Taeniopygia guttata* | Ensembl Vertebrates 62 | Birds |
| AP | *Tetraodon nigroviridis* | Ensembl Vertebrates 62 | Fishes |
| UE | *Thalassiosira pseudonana* | Ensembl Protists 9 | Heterokonta |
|  | *Tribolium castaneum* | -1- | Invertebrates |
| AP RP | *Zea mays* | Ensembl Plants 9 | Plants |
