# Supplementary material for: Neutral Theory Predicts the Relative Abundance and Diversity of Genetic Elements in a Broad Array of Eukaryotic Genomes
Source: PLoS One. 2013 Jun 14;8(6):e63915. doi: 10.1371/journal.pone.0063915 (PMC3683013; doi:10.1371/journal.pone.0063915)
Supplement: Table S2 — Genetic species and number of elements in two selected chromosomes. Genetic species are arranged according to the observed ranking of abundance. (DOCX) [file pone.0063915.s004.docx]

| ***Homo sapiens chromosome 1*** | | | | | | | |
| --- | --- | --- | --- | --- | --- | --- | --- |
| ***Biotypes*** | | ***Repeated Classes (cont.)*** | | | | | |
| Protein coding | 2,032 | DNA/TcMar-Tigger | | 4,274 | | DNA/MuDR | 90 |
| processed transcript | 1,055 | LINE/CR1 | | 2,920 | | srpRNA | 82 |
| IG V | 221 | DNA/hAT-Tip100 | | 1,309 | | LTR | 81 |
| miRNA | 145 | DNA/hAT-Blackjack | | 770 | | rRNA | 65 |
| lincRNA | 134 | DNA/TcMar-Mariner | | 583 | | Satellite | 65 |
| snRNA | 128 | LINE/RTE-X | | 580 | | scRNA | 56 |
| snoRNA | 106 | LTR/Gypsy | | 572 | | RC/Helitron | 53 |
| miscRNA | 66 | DNA/hAT | | 502 | | LINE/RTE-BovB | 27 |
| ***Repeated Classes*** | | LTR/ERVK | | 444 | | RNA | 23 |
| SINE/Alu | 51,377 | DNA/TcMar-Tc2 | | 314 | | SINE | 23 |
| LINE/L1 | 34,418 | Unknown | | 252 | | LINE/Dong-R4 | 20 |
| Simple Repeat | 31,504 | Other | | 223 | | Satellite/centr | 16 |
| Low complexity | 29,261 | snRNA | | 202 | | LTR/ERV | 13 |
| SINE/MIR | 28,046 | DNA | | 143 | | LINE/RTE | 6 |
| LINE/L2 | 22,668 | DNA/TcMar | | 132 | | DNA/Merlin | ***3*** |
| LTR/ERVL-MaLR | 12,156 | SINE/tRNA | | 125 | | DNA/TcMar-Pogo | ***2*** |
| DNA/hAT-Charlie | 8,900 | pseudo-tRNA | | 117 | | Satellite/telo | 2 |
| LTR/ERV1 | 6,763 | SINE/Deu | | 109 | | LINE/Penelope | 2 |
| LTR/ERVL | 5,671 | DNA/PiggyBac | | 97 | | Satellite/acro | 2 |
|  | | | | | | | |
| ***Drosophila melanogaster chromosome 2L*** | | | | | | | |
| **Biotypes** | | ***Repeated Classes (cont.)*** | | | | | |
| Protein coding | 2,601 | RC/Helitron | 431 | | LTR/Copia | | 27 |
| tRNA | 41 | LTR/Gypsy | 287 | | LINE/R1 | | 24 |
| snoRNA | 39 | LTR/Pao | 133 | | LINE/I | | 21 |
| snRNA | 32 | LINE/Jockey | 111 | | DNA/hAT-hobo | | 16 |
| miRNA | 21 | DNA/P | 87 | | Unknown | | 15 |
| ncRNA | 18 | Satellite | 68 | | LINE/LOA | | 7 |
| **Repeated Classes** | | DNA/TcMar-Tc1 | 51 | | DNA/TcMar-Pogo | | 6 |
| Low_complexity | 6,263 | DNA/Transib | 32 | | DNA/PiggyBac | | 1 |
| Simple_repeat | 3,946 | LINE/CR1 | 32 | |  | |  |
