# Supplementary material for: Neutral Theory Predicts the Relative Abundance and Diversity of Genetic Elements in a Broad Array of Eukaryotic Genomes
Source: PLoS One. 2013 Jun 14;8(6):e63915. doi: 10.1371/journal.pone.0063915 (PMC3683013; doi:10.1371/journal.pone.0063915)
Supplement: Table S3 — Optimization of Models and Parameters. The columns “theta Etienne” and “theta Ewens” show the values of θ calculated according to Etienne model Ewens sampling formula, respectively. The “m” column shows the migration parameter according to Etienne's model. “Best model” column shows which model was found to have a better fit for each chromosome. “lnL p(q)-val” column corresponds to the p-value (q-value) of the neutrality test comparing the likelihood to fit Etienne's model of simulated data to the likelihood of observed data. “N” column shows the number of simulations that where generated for each chromosome (in the context of the neutrality test comparing likelihoods). “delta-H” represents the difference in shannon entropy (H) between 1,000 simulation and the observed values. “shannon p(q)-val” column shows the p-value(q-value) of the neutrality test that consists in comparing the shannon entropy of simulated distribution of species' abundances to the observed data. (DOCX) [file pone.0063915.s005.docx]

|  |  |  |  |  |  |  |  | **Neutrality test** | | | |
| --- | --- | --- | --- | --- | --- | --- | --- | --- | --- | --- | --- |
|  |  |  |  |  |  |  |  | **Comparison of likelihoods** | | **Comparison of entropies** | |
| **Species** | **Ch** | **J** | **S** | **theta Etienne** | **theta Ewens** | **m** | **Best model** | **lnL p(q)-val** | **N** | **delta-H** | **shannon p(q)-val** |
| Drosophila_melanogaster | 2R | 13630 | 27 | 4.003 | 3.164 | --- | ewens | 0.3 (0.582) | 100 | -0.163 | 0.297 (0.659) |
| Drosophila_melanogaster | 3R | 17182 | 24 | 3.031 | 2.677 | --- | ewens | 0.25 (0.582) | 100 | -0.409 | 0.139 (0.659) |
| Drosophila_melanogaster | 4 | 1481 | 16 | 2.621 | 2.408 | --- | ewens | 0.4 (0.582) | 100 | -0.062 | 0.398 (0.659) |
| Drosophila_melanogaster | 3L | 16564 | 27 | 3.691 | 3.082 | --- | ewens | 0.27 (0.582) | 100 | -0.327 | 0.171 (0.659) |
| Drosophila_melanogaster | 2L | 14310 | 25 | 3.624 | 2.875 | 0.079 | etienne | 0.37 (0.582) | 100 | -0.471 | 0.096 (0.659) |
| Drosophila_melanogaster | X | 20787 | 26 | 3.151 | 2.865 | --- | ewens | 0.15 (0.582) | 100 | -0.51 | 0.09 (0.659) |
| Oryzias_latipes | 24 | 7345 | 15 | 1.946 | 1.73 | --- | ewens | 0.43 (0.582) | 100 | -0.012 | 0.453 (0.659) |
| Oryzias_latipes | 20 | 7564 | 18 | 2.145 | 2.137 | --- | ewens | 0.4 (0.582) | 100 | -0.213 | 0.269 (0.659) |
| Oryzias_latipes | 21 | 9883 | 18 | 2.151 | 2.06 | --- | ewens | 0.41 (0.582) | 100 | -0.127 | 0.342 (0.659) |
| Oryzias_latipes | 22 | 9004 | 22 | 2.674 | 2.639 | --- | ewens | 0.16 (0.582) | 100 | -0.305 | 0.192 (0.659) |
| Oryzias_latipes | 23 | 7321 | 18 | 2.174 | 2.147 | --- | ewens | 0.35 (0.582) | 100 | -0.161 | 0.308 (0.659) |
| Oryzias_latipes | 1 | 11158 | 21 | 2.486 | 2.427 | --- | ewens | 0.3 (0.582) | 100 | -0.227 | 0.257 (0.659) |
| Oryzias_latipes | 3 | 11937 | 21 | 2.405 | 2.405 | --- | ewens | 0.33 (0.582) | 100 | -0.293 | 0.207 (0.659) |
| Oryzias_latipes | 2 | 7053 | 20 | 2.465 | 2.443 | --- | ewens | 0.3 (0.582) | 100 | -0.299 | 0.197 (0.659) |
| Oryzias_latipes | 5 | 10548 | 20 | 2.31 | 2.31 | --- | ewens | 0.36 (0.582) | 100 | -0.21 | 0.273 (0.659) |
| Oryzias_latipes | 4 | 11361 | 19 | 2.154 | 2.154 | --- | ewens | 0.49 (0.587) | 100 | -0.156 | 0.315 (0.659) |
| Oryzias_latipes | 7 | 9956 | 21 | 2.465 | 2.465 | --- | ewens | 0.24 (0.582) | 100 | -0.293 | 0.206 (0.659) |
| Oryzias_latipes | 6 | 9089 | 19 | 2.299 | 2.22 | --- | ewens | 0.31 (0.582) | 100 | -0.211 | 0.271 (0.659) |
| Oryzias_latipes | 9 | 10618 | 19 | 2.174 | 2.174 | --- | ewens | 0.33 (0.582) | 100 | -0.197 | 0.282 (0.659) |
| Oryzias_latipes | 8 | 8395 | 21 | 2.524 | 2.524 | --- | ewens | 0.2 (0.582) | 100 | -0.308 | 0.197 (0.659) |
| Oryzias_latipes | 11 | 9268 | 19 | 2.463 | 2.214 | --- | ewens | 0.33 (0.582) | 100 | -0.173 | 0.3 (0.659) |
| Oryzias_latipes | 10 | 8787 | 18 | 2.282 | 2.093 | --- | ewens | 0.32 (0.582) | 100 | -0.197 | 0.284 (0.659) |
| Oryzias_latipes | 13 | 10901 | 21 | 2.517 | 2.434 | --- | ewens | 0.25 (0.582) | 100 | -0.22 | 0.259 (0.659) |
| Oryzias_latipes | 12 | 10030 | 16 | 2.075 | 1.791 | --- | ewens | 0.36 (0.582) | 100 | -0.056 | 0.413 (0.659) |
| Oryzias_latipes | 15 | 9233 | 19 | 2.215 | 2.215 | --- | ewens | 0.26 (0.582) | 100 | -0.239 | 0.248 (0.659) |
| Oryzias_latipes | 14 | 9368 | 19 | 2.491 | 2.211 | --- | ewens | 0.32 (0.582) | 100 | -0.181 | 0.296 (0.659) |
| Oryzias_latipes | 17 | 10581 | 18 | 2.098 | 2.041 | --- | ewens | 0.58 (0.62) | 100 | -0.084 | 0.385 (0.659) |
| Oryzias_latipes | 16 | 9747 | 18 | 2.183 | 2.064 | --- | ewens | 0.34 (0.582) | 100 | -0.16 | 0.312 (0.659) |
| Oryzias_latipes | 19 | 7223 | 21 | 2.579 | 2.578 | --- | ewens | 0.17 (0.582) | 100 | -0.344 | 0.171 (0.659) |
| Oryzias_latipes | 18 | 8692 | 17 | 2.354 | 1.96 | --- | ewens | 0.44 (0.582) | 100 | -0.027 | 0.439 (0.659) |
| Monodelphis_domestica | 1 | 933209 | 44 | 4.406 | 3.478 | 0.024 | etienne | 0.3 (0.582) | 50 | -0.017 | 0.44 (0.659) |
| Monodelphis_domestica | 3 | 643466 | 44 | 4.389 | 3.595 | 0.041 | etienne | 0.25 (0.582) | 28 | 0.013 | 0.48 (0.671) |
| Monodelphis_domestica | 2 | 675788 | 44 | 4.461 | 3.579 | 0.032 | etienne | 0.105 (0.582) | 19 | -0.02 | 0.435 (0.659) |
| Monodelphis_domestica | 5 | 363398 | 44 | 4.333 | 3.79 | --- | ewens | 0.329 (0.582) | 70 | 0.13 | 0.621 (0.737) |
| Monodelphis_domestica | 4 | 532447 | 43 | 4.388 | 3.565 | 0.039 | etienne | 0.311 (0.582) | 61 | 0.001 | 0.461 (0.66) |
| Monodelphis_domestica | 7 | 306906 | 42 | 4.407 | 3.659 | 0.058 | etienne | 0.391 (0.582) | 87 | 0.005 | 0.469 (0.664) |
| Monodelphis_domestica | 6 | 363771 | 43 | 4.401 | 3.695 | 0.064 | etienne | 0.39 (0.582) | 77 | -0.017 | 0.441 (0.659) |
| Monodelphis_domestica | X | 84888 | 41 | 4.317 | 4.07 | --- | ewens | 0.47 (0.584) | 100 | 0.036 | 0.507 (0.682) |
| Monodelphis_domestica | 8 | 373826 | 43 | 4.389 | 3.685 | 0.064 | etienne | 0.407 (0.582) | 81 | -0.005 | 0.458 (0.659) |
| Plasmodium_falciparum | 2 | 5910 | 7 | 0.709 | 0.708 | --- | ewens | 0.42 (0.582) | 100 | 0.078 | 0.56 (0.713) |
| Plasmodium_falciparum | 3 | 6529 | 8 | 0.826 | 0.825 | --- | ewens | 0.32 (0.582) | 100 | 0 | 0.486 (0.672) |
| Plasmodium_falciparum | 13 | 18738 | 10 | 0.953 | 0.953 | --- | ewens | 0.34 (0.582) | 100 | -0.111 | 0.386 (0.659) |
| Plasmodium_falciparum | 12 | 14462 | 7 | 0.694 | 0.637 | --- | ewens | 0.54 (0.601) | 100 | 0.137 | 0.614 (0.736) |
| Plasmodium_falciparum | 6 | 8852 | 8 | 0.795 | 0.795 | --- | ewens | 0.43 (0.582) | 100 | 0.045 | 0.526 (0.688) |
| Plasmodium_falciparum | 7 | 9057 | 9 | 0.99 | 0.915 | --- | ewens | 0.37 (0.582) | 100 | -0.061 | 0.428 (0.659) |
| Plasmodium_falciparum | 4 | 7149 | 7 | 0.776 | 0.692 | --- | ewens | 0.49 (0.587) | 100 | 0.093 | 0.573 (0.713) |
| Plasmodium_falciparum | 10 | 10764 | 6 | 0.688 | 0.543 | --- | ewens | 0.58 (0.62) | 100 | 0.228 | 0.698 (0.797) |
| Plasmodium_falciparum | 8 | 8841 | 8 | 0.837 | 0.795 | --- | ewens | 0.46 (0.582) | 100 | 0.023 | 0.506 (0.682) |
| Plasmodium_falciparum | 9 | 10197 | 6 | 0.67 | 0.546 | --- | ewens | 0.54 (0.601) | 100 | 0.222 | 0.688 (0.794) |
| Plasmodium_falciparum | 1 | 3881 | 8 | 0.983 | 0.884 | --- | ewens | 0.42 (0.582) | 100 | 0.002 | 0.482 (0.671) |
| Plasmodium_falciparum | 11 | 13360 | 9 | 0.873 | 0.873 | --- | ewens | 0.33 (0.582) | 100 | -0.053 | 0.435 (0.659) |
| Plasmodium_falciparum | 5 | 8550 | 8 | 0.817 | 0.798 | --- | ewens | 0.35 (0.582) | 100 | -0.007 | 0.478 (0.671) |
| Plasmodium_falciparum | 14 | 21827 | 7 | 0.627 | 0.609 | --- | ewens | 0.44 (0.582) | 100 | 0.131 | 0.611 (0.736) |
| Oryza_sativa | 1 | 34632 | 26 | 4.278 | 2.692 | 0.01 | etienne | 0.74 (0.747) | 100 | 0.262 | 0.793 (0.839) |
| Oryza_sativa | 3 | 29312 | 26 | 4.184 | 2.746 | 0.015 | etienne | 0.6 (0.627) | 100 | 0.26 | 0.781 (0.838) |
| Oryza_sativa | 2 | 28787 | 27 | 4.274 | 2.874 | 0.018 | etienne | 0.6 (0.627) | 100 | 0.244 | 0.772 (0.836) |
| Oryza_sativa | 5 | 23823 | 25 | 4.192 | 2.692 | 0.014 | etienne | 0.68 (0.69) | 100 | 0.273 | 0.799 (0.839) |
| Oryza_sativa | 4 | 26213 | 26 | 4.12 | 2.784 | 0.019 | etienne | 0.66 (0.671) | 100 | 0.278 | 0.804 (0.839) |
| Oryza_sativa | 7 | 23668 | 25 | 4.384 | 2.695 | 0.011 | etienne | 0.61 (0.635) | 100 | 0.279 | 0.812 (0.839) |
| Oryza_sativa | 6 | 25697 | 27 | 4.39 | 2.914 | 0.017 | etienne | 0.71 (0.719) | 100 | 0.253 | 0.778 (0.836) |
| Oryza_sativa | 9 | 18882 | 27 | 4.565 | 3.03 | 0.02 | etienne | 0.64 (0.655) | 100 | 0.256 | 0.787 (0.839) |
| Oryza_sativa | 8 | 22756 | 25 | 4.291 | 2.708 | 0.013 | etienne | 0.79 (0.796) | 100 | 0.286 | 0.81 (0.839) |
| Arabidopsis_thaliana | 1 | 21841 | 31 | 4.675 | 3.486 | 0.045 | etienne | 0.44 (0.582) | 100 | -0.393 | 0.11 (0.659) |
| Arabidopsis_thaliana | 3 | 16815 | 33 | 4.642 | 3.878 | --- | ewens | 0.26 (0.582) | 100 | -0.181 | 0.264 (0.659) |
| Arabidopsis_thaliana | 2 | 14845 | 31 | 4.757 | 3.67 | 0.067 | etienne | 0.43 (0.582) | 100 | -0.339 | 0.136 (0.659) |
| Arabidopsis_thaliana | 5 | 19372 | 31 | 4.59 | 3.541 | 0.061 | etienne | 0.38 (0.582) | 100 | -0.436 | 0.094 (0.659) |
| Arabidopsis_thaliana | 4 | 13396 | 31 | 4.903 | 3.722 | 0.062 | etienne | 0.36 (0.582) | 100 | -0.337 | 0.14 (0.659) |
| Pan_troglodytes | 20 | 67524 | 51 | 6.2 | 5.346 | --- | ewens | 0.3 (0.582) | 100 | 0.076 | 0.572 (0.713) |
| Pan_troglodytes | 21 | 33670 | 49 | 6.073 | 5.568 | --- | ewens | 0.12 (0.582) | 100 | -0.008 | 0.456 (0.659) |
| Pan_troglodytes | 22 | 39799 | 50 | 6.18 | 5.577 | --- | ewens | 0.18 (0.582) | 100 | -0.126 | 0.297 (0.659) |
| Pan_troglodytes | 1 | 239915 | 55 | 6.336 | 5.06 | 0.044 | etienne | 0.34 (0.582) | 100 | -0.103 | 0.327 (0.659) |
| Pan_troglodytes | 3 | 201173 | 55 | 6.31 | 5.153 | 0.059 | etienne | 0.2 (0.582) | 100 | -0.074 | 0.359 (0.659) |
| Pan_troglodytes | 5 | 177208 | 55 | 6.498 | 5.223 | 0.051 | etienne | 0.24 (0.582) | 100 | -0.106 | 0.319 (0.659) |
| Pan_troglodytes | 4 | 188660 | 55 | 6.472 | 5.188 | 0.049 | etienne | 0.34 (0.582) | 100 | -0.108 | 0.316 (0.659) |
| Pan_troglodytes | 7 | 158101 | 52 | 6.405 | 4.965 | 0.037 | etienne | 0.36 (0.582) | 100 | -0.132 | 0.29 (0.659) |
| Pan_troglodytes | 6 | 166787 | 54 | 6.395 | 5.15 | 0.053 | etienne | 0.37 (0.582) | 100 | -0.092 | 0.34 (0.659) |
| Pan_troglodytes | 9 | 117267 | 53 | 6.335 | 5.24 | 0.077 | etienne | 0.23 (0.582) | 100 | -0.105 | 0.323 (0.659) |
| Pan_troglodytes | 8 | 142767 | 53 | 6.471 | 5.128 | 0.047 | etienne | 0.24 (0.582) | 100 | -0.103 | 0.327 (0.659) |
| Pan_troglodytes | Y | 20688 | 47 | 5.667 | 5.667 | --- | ewens | 0.22 (0.582) | 100 | -0.096 | 0.341 (0.659) |
| Pan_troglodytes | X | 135854 | 53 | 6.375 | 5.156 | 0.059 | etienne | 0.28 (0.582) | 100 | -0.132 | 0.293 (0.659) |
| Pan_troglodytes | 11 | 131482 | 54 | 6.56 | 5.284 | 0.057 | etienne | 0.29 (0.582) | 100 | -0.115 | 0.308 (0.659) |
| Pan_troglodytes | 10 | 131426 | 53 | 6.344 | 5.175 | 0.065 | etienne | 0.25 (0.582) | 100 | -0.118 | 0.313 (0.659) |
| Pan_troglodytes | 13 | 88195 | 54 | 6.389 | 5.527 | 0.135 | etienne | 0.2 (0.582) | 100 | -0.137 | 0.287 (0.659) |
| Pan_troglodytes | 12 | 142252 | 53 | 6.486 | 5.13 | 0.046 | etienne | 0.17 (0.582) | 100 | -0.111 | 0.309 (0.659) |
| Pan_troglodytes | 15 | 82280 | 52 | 6.284 | 5.339 | 0.112 | etienne | 0.27 (0.582) | 100 | -0.12 | 0.303 (0.659) |
| Pan_troglodytes | 14 | 90667 | 53 | 6.221 | 5.394 | 0.138 | etienne | 0.37 (0.582) | 100 | -0.077 | 0.36 (0.659) |
| Pan_troglodytes | 17 | 89414 | 52 | 6.122 | 5.288 | --- | ewens | 0.21 (0.582) | 100 | -0.064 | 0.376 (0.659) |
| Pan_troglodytes | 16 | 90184 | 53 | 6.462 | 5.398 | 0.092 | etienne | 0.22 (0.582) | 100 | -0.194 | 0.227 (0.659) |
| Pan_troglodytes | 19 | 70994 | 52 | 6.132 | 5.432 | --- | ewens | 0.13 (0.582) | 100 | -0.215 | 0.214 (0.659) |
| Pan_troglodytes | 18 | 73835 | 53 | 6.315 | 5.524 | 0.159 | etienne | 0.29 (0.582) | 100 | -0.104 | 0.328 (0.659) |
| Pan_troglodytes | 2a | 109367 | 52 | 6.21 | 5.169 | 0.084 | etienne | 0.25 (0.582) | 100 | -0.081 | 0.355 (0.659) |
| Pan_troglodytes | 2b | 126685 | 54 | 6.577 | 5.306 | 0.059 | etienne | 0.3 (0.582) | 100 | -0.115 | 0.311 (0.659) |
| Homo_sapiens | 20 | 69222 | 52 | 6.542 | 5.448 | 0.095 | etienne | 0.26 (0.582) | 100 | -0.099 | 0.328 (0.659) |
| Homo_sapiens | 21 | 36427 | 53 | 6.721 | 6.028 | --- | ewens | 0.21 (0.582) | 100 | -0.04 | 0.41 (0.659) |
| Homo_sapiens | 22 | 42461 | 54 | 6.795 | 6.038 | --- | ewens | 0.12 (0.582) | 100 | -0.161 | 0.266 (0.659) |
| Homo_sapiens | 1 | 248949 | 56 | 6.738 | 5.143 | 0.028 | etienne | 0.31 (0.582) | 100 | -0.13 | 0.291 (0.659) |
| Homo_sapiens | 3 | 200776 | 54 | 6.487 | 5.05 | 0.036 | etienne | 0.35 (0.582) | 100 | -0.084 | 0.352 (0.659) |
| Homo_sapiens | 2 | 240748 | 57 | 6.803 | 5.264 | 0.032 | etienne | 0.32 (0.582) | 100 | -0.132 | 0.292 (0.659) |
| Homo_sapiens | 5 | 178768 | 54 | 6.532 | 5.112 | 0.039 | etienne | 0.29 (0.582) | 100 | -0.095 | 0.331 (0.659) |
| Homo_sapiens | 4 | 190798 | 55 | 6.555 | 5.182 | 0.042 | etienne | 0.26 (0.582) | 100 | -0.099 | 0.328 (0.659) |
| Homo_sapiens | 7 | 162848 | 57 | 6.947 | 5.485 | 0.044 | etienne | 0.23 (0.582) | 100 | -0.199 | 0.212 (0.659) |
| Homo_sapiens | 6 | 168606 | 54 | 6.569 | 5.144 | 0.04 | etienne | 0.31 (0.582) | 100 | -0.103 | 0.321 (0.659) |
| Homo_sapiens | 9 | 128726 | 54 | 6.662 | 5.296 | 0.05 | etienne | 0.24 (0.582) | 100 | -0.135 | 0.289 (0.659) |
| Homo_sapiens | 8 | 146967 | 52 | 6.429 | 5.004 | 0.039 | etienne | 0.39 (0.582) | 100 | -0.077 | 0.356 (0.659) |
| Homo_sapiens | Y | 21960 | 48 | 5.866 | 5.758 | --- | ewens | 0.17 (0.582) | 100 | -0.099 | 0.337 (0.659) |
| Homo_sapiens | X | 153676 | 54 | 6.468 | 5.195 | 0.053 | etienne | 0.26 (0.582) | 100 | -0.141 | 0.277 (0.659) |
| Homo_sapiens | 11 | 137951 | 54 | 6.672 | 5.256 | 0.045 | etienne | 0.27 (0.582) | 100 | -0.111 | 0.307 (0.659) |
| Homo_sapiens | 10 | 137393 | 53 | 6.509 | 5.15 | 0.047 | etienne | 0.38 (0.582) | 100 | -0.114 | 0.313 (0.659) |
| Homo_sapiens | 13 | 96467 | 54 | 6.568 | 5.47 | 0.088 | etienne | 0.22 (0.582) | 100 | -0.131 | 0.288 (0.659) |
| Homo_sapiens | 12 | 143743 | 53 | 6.559 | 5.124 | 0.041 | etienne | 0.32 (0.582) | 100 | -0.107 | 0.317 (0.659) |
| Homo_sapiens | 15 | 86781 | 54 | 6.457 | 5.537 | 0.123 | etienne | 0.37 (0.582) | 100 | -0.145 | 0.276 (0.659) |
| Homo_sapiens | 14 | 92373 | 58 | 7.223 | 5.958 | 0.082 | etienne | 0.15 (0.582) | 100 | -0.192 | 0.215 (0.659) |
| Homo_sapiens | 17 | 93105 | 52 | 6.515 | 5.264 | 0.065 | etienne | 0.23 (0.582) | 100 | -0.24 | 0.182 (0.659) |
| Homo_sapiens | 16 | 96096 | 54 | 6.737 | 5.472 | 0.068 | etienne | 0.23 (0.582) | 100 | -0.229 | 0.189 (0.659) |
| Homo_sapiens | 19 | 76175 | 52 | 6.129 | 5.387 | --- | ewens | 0.22 (0.582) | 100 | -0.194 | 0.237 (0.659) |
| Homo_sapiens | 18 | 74089 | 54 | 6.498 | 5.64 | 0.146 | etienne | 0.23 (0.582) | 100 | -0.126 | 0.299 (0.659) |
| Arabidopsis_lyrata | 1 | 25822 | 25 | 3.93 | 2.666 | 0.019 | etienne | 0.48 (0.586) | 100 | -0.25 | 0.213 (0.659) |
| Arabidopsis_lyrata | 3 | 19332 | 25 | 4.039 | 2.764 | 0.023 | etienne | 0.51 (0.593) | 100 | -0.258 | 0.203 (0.659) |
| Arabidopsis_lyrata | 2 | 15451 | 25 | 4.089 | 2.846 | 0.029 | etienne | 0.41 (0.582) | 100 | -0.299 | 0.173 (0.659) |
| Arabidopsis_lyrata | 5 | 17265 | 25 | 4.136 | 2.805 | 0.023 | etienne | 0.4 (0.582) | 100 | -0.273 | 0.186 (0.659) |
| Arabidopsis_lyrata | 4 | 18220 | 26 | 4.189 | 2.914 | 0.027 | etienne | 0.41 (0.582) | 100 | -0.282 | 0.182 (0.659) |
| Arabidopsis_lyrata | 7 | 19450 | 25 | 3.922 | 2.762 | 0.028 | etienne | 0.51 (0.593) | 100 | -0.287 | 0.184 (0.659) |
| Arabidopsis_lyrata | 6 | 19601 | 25 | 3.864 | 2.759 | 0.031 | etienne | 0.49 (0.587) | 100 | -0.268 | 0.199 (0.659) |
| Arabidopsis_lyrata | 8 | 18013 | 24 | 3.631 | 2.661 | 0.038 | etienne | 0.47 (0.584) | 100 | -0.245 | 0.215 (0.659) |
| Caenorhabditis_elegans | I | 16671 | 33 | 4.478 | 3.883 | 0.182 | etienne | 0.41 (0.582) | 100 | -0.198 | 0.242 (0.659) |
| Caenorhabditis_elegans | IV | 31715 | 31 | 3.837 | 3.326 | --- | ewens | 0.32 (0.582) | 100 | -0.318 | 0.17 (0.659) |
| Caenorhabditis_elegans | II | 15979 | 31 | 4.204 | 3.633 | --- | ewens | 0.37 (0.582) | 100 | -0.039 | 0.416 (0.659) |
| Caenorhabditis_elegans | V | 20974 | 31 | 4.544 | 3.504 | 0.059 | etienne | 0.51 (0.593) | 100 | -0.114 | 0.328 (0.659) |
| Caenorhabditis_elegans | X | 14878 | 30 | 30 | 3.532 | --- | ewens | 0.42 (0.582) | 100 | 0.052 | 0.524 (0.688) |
| Caenorhabditis_elegans | III | 15813 | 29 | 3.858 | 3.368 | --- | ewens | 0.55 (0.604) | 100 | 0.063 | 0.535 (0.691) |
| Canis_familiaris | 24 | 60334 | 45 | 4.702 | 4.702 | --- | ewens | 0.47 (0.584) | 100 | 0.081 | 0.572 (0.713) |
| Canis_familiaris | 25 | 59071 | 46 | 5.16 | 4.833 | --- | ewens | 0.23 (0.582) | 100 | -0.011 | 0.448 (0.659) |
| Canis_familiaris | 26 | 48508 | 41 | 4.725 | 4.343 | --- | ewens | 0.36 (0.582) | 100 | 0.066 | 0.54 (0.695) |
| Canis_familiaris | 27 | 57509 | 46 | 5.206 | 4.848 | --- | ewens | 0.21 (0.582) | 100 | -0.054 | 0.391 (0.659) |
| Canis_familiaris | 20 | 72730 | 45 | 4.784 | 4.601 | --- | ewens | 0.34 (0.582) | 100 | 0.059 | 0.538 (0.694) |
| Canis_familiaris | 21 | 63148 | 44 | 4.894 | 4.56 | --- | ewens | 0.26 (0.582) | 100 | 0.026 | 0.493 (0.673) |
| Canis_familiaris | 22 | 70677 | 45 | 5.196 | 4.616 | --- | ewens | 0.23 (0.582) | 100 | -0.035 | 0.42 (0.659) |
| Canis_familiaris | 23 | 61674 | 44 | 5.038 | 4.572 | --- | ewens | 0.37 (0.582) | 100 | 0.079 | 0.57 (0.713) |
| Canis_familiaris | 28 | 46691 | 44 | 4.728 | 4.727 | --- | ewens | 0.3 (0.582) | 100 | 0.021 | 0.488 (0.672) |
| Canis_familiaris | 29 | 49261 | 44 | 5.168 | 4.697 | --- | ewens | 0.29 (0.582) | 100 | -0.013 | 0.444 (0.659) |
| Canis_familiaris | 1 | 144103 | 47 | 5.281 | 4.478 | 0.093 | etienne | 0.3 (0.582) | 100 | -0.103 | 0.329 (0.659) |
| Canis_familiaris | 3 | 108375 | 46 | 5.129 | 4.509 | 0.151 | etienne | 0.28 (0.582) | 100 | -0.074 | 0.362 (0.659) |
| Canis_familiaris | 2 | 103241 | 48 | 5.114 | 4.755 | --- | ewens | 0.25 (0.582) | 100 | 0.002 | 0.463 (0.66) |
| Canis_familiaris | 5 | 105729 | 48 | 5.119 | 4.743 | --- | ewens | 0.34 (0.582) | 100 | 0.032 | 0.508 (0.682) |
| Canis_familiaris | 4 | 107872 | 48 | 5.27 | 4.732 | --- | ewens | 0.26 (0.582) | 100 | 0.022 | 0.491 (0.673) |
| Canis_familiaris | 7 | 93993 | 46 | 5.234 | 4.581 | --- | ewens | 0.38 (0.582) | 100 | 0.042 | 0.514 (0.684) |
| Canis_familiaris | 6 | 96674 | 45 | 5.095 | 4.455 | 0.144 | etienne | 0.29 (0.582) | 100 | -0.035 | 0.415 (0.659) |
| Canis_familiaris | 9 | 78273 | 44 | 5.021 | 4.448 | 0.175 | etienne | 0.19 (0.582) | 100 | -0.086 | 0.353 (0.659) |
| Canis_familiaris | 8 | 88686 | 47 | 5.35 | 4.724 | --- | ewens | 0.25 (0.582) | 100 | 0.02 | 0.487 (0.672) |
| Canis_familiaris | 12 | 88759 | 45 | 5.16 | 4.498 | --- | ewens | 0.31 (0.582) | 100 | 0.038 | 0.514 (0.684) |
| Canis_familiaris | X | 150013 | 48 | 5.015 | 4.566 | --- | ewens | 0.35 (0.582) | 100 | -0.025 | 0.427 (0.659) |
| Canis_familiaris | 11 | 89937 | 45 | 5.062 | 4.491 | --- | ewens | 0.28 (0.582) | 100 | 0.048 | 0.522 (0.688) |
| Canis_familiaris | 10 | 83296 | 46 | 5.161 | 4.644 | --- | ewens | 0.31 (0.582) | 100 | 0.038 | 0.511 (0.684) |
| Canis_familiaris | 13 | 76681 | 47 | 5.099 | 4.803 | --- | ewens | 0.23 (0.582) | 100 | 0.007 | 0.471 (0.665) |
| Canis_familiaris | 38 | 29202 | 42 | 5.04 | 4.756 | --- | ewens | 0.21 (0.582) | 100 | -0.011 | 0.449 (0.659) |
| Canis_familiaris | 15 | 81374 | 45 | 5.04 | 4.542 | --- | ewens | 0.38 (0.582) | 100 | 0.035 | 0.511 (0.684) |
| Canis_familiaris | 14 | 70591 | 44 | 5.124 | 4.501 | 0.162 | etienne | 0.4 (0.582) | 100 | -0.077 | 0.361 (0.659) |
| Canis_familiaris | 17 | 76309 | 45 | 5.146 | 4.575 | 0.185 | etienne | 0.46 (0.582) | 100 | -0.058 | 0.386 (0.659) |
| Canis_familiaris | 16 | 67510 | 44 | 5.092 | 4.524 | --- | ewens | 0.35 (0.582) | 100 | -0.004 | 0.457 (0.659) |
| Canis_familiaris | 19 | 64547 | 45 | 5.141 | 4.665 | --- | ewens | 0.3 (0.582) | 100 | -0.026 | 0.429 (0.659) |
| Canis_familiaris | 18 | 67006 | 43 | 4.952 | 4.412 | --- | ewens | 0.35 (0.582) | 100 | 0.05 | 0.524 (0.688) |
| Canis_familiaris | 31 | 46450 | 43 | 5.016 | 4.608 | --- | ewens | 0.26 (0.582) | 100 | -0.041 | 0.408 (0.659) |
| Canis_familiaris | 30 | 49416 | 42 | 4.897 | 4.453 | --- | ewens | 0.32 (0.582) | 100 | 0.044 | 0.522 (0.688) |
| Canis_familiaris | 37 | 35838 | 43 | 4.93 | 4.759 | --- | ewens | 0.35 (0.582) | 100 | 0.004 | 0.477 (0.671) |
| Canis_familiaris | 36 | 35733 | 42 | 4.987 | 4.635 | --- | ewens | 0.24 (0.582) | 100 | -0.014 | 0.442 (0.659) |
| Canis_familiaris | 35 | 31042 | 42 | 5.034 | 4.718 | --- | ewens | 0.27 (0.582) | 100 | -0.01 | 0.446 (0.659) |
| Canis_familiaris | 34 | 47240 | 43 | 5.125 | 4.599 | --- | ewens | 0.29 (0.582) | 100 | 0.027 | 0.5 (0.678) |
| Canis_familiaris | 33 | 37704 | 45 | 5.317 | 4.98 | --- | ewens | 0.13 (0.582) | 100 | -0.059 | 0.384 (0.659) |
| Canis_familiaris | 32 | 48065 | 46 | 5.182 | 4.955 | --- | ewens | 0.19 (0.582) | 100 | -0.074 | 0.366 (0.659) |
| Macaca_mulatta | 11 | 141974 | 50 | 6.009 | 4.807 | 0.051 | etienne | 0.34 (0.582) | 100 | -0.064 | 0.375 (0.659) |
| Macaca_mulatta | 10 | 104699 | 51 | 5.976 | 5.082 | 0.106 | etienne | 0.21 (0.582) | 100 | -0.114 | 0.316 (0.659) |
| Macaca_mulatta | 13 | 129177 | 51 | 6.114 | 4.966 | 0.061 | etienne | 0.27 (0.582) | 100 | -0.083 | 0.346 (0.659) |
| Macaca_mulatta | 12 | 101170 | 50 | 6.083 | 4.99 | 0.072 | etienne | 0.37 (0.582) | 100 | -0.066 | 0.373 (0.659) |
| Macaca_mulatta | 20 | 83583 | 50 | 5.984 | 5.099 | --- | ewens | 0.32 (0.582) | 100 | -0.009 | 0.45 (0.659) |
| Macaca_mulatta | 14 | 128405 | 52 | 6.141 | 5.078 | 0.075 | etienne | 0.3 (0.582) | 100 | -0.072 | 0.367 (0.659) |
| Macaca_mulatta | 17 | 90493 | 52 | 6.146 | 5.281 | 0.124 | etienne | 0.15 (0.582) | 100 | -0.102 | 0.323 (0.659) |
| Macaca_mulatta | 16 | 84626 | 50 | 5.949 | 5.092 | 0.119 | etienne | 0.22 (0.582) | 100 | -0.195 | 0.23 (0.659) |
| Macaca_mulatta | 19 | 62878 | 49 | 5.577 | 5.153 | --- | ewens | 0.27 (0.582) | 100 | -0.173 | 0.264 (0.659) |
| Macaca_mulatta | 18 | 69811 | 50 | 5.953 | 5.207 | 0.159 | etienne | 0.26 (0.582) | 100 | -0.066 | 0.375 (0.659) |
| Macaca_mulatta | 15 | 112958 | 51 | 5.848 | 5.04 | 0.121 | etienne | 0.27 (0.582) | 100 | -0.053 | 0.389 (0.659) |
| Macaca_mulatta | 1 | 238823 | 52 | 6.114 | 4.756 | 0.033 | etienne | 0.31 (0.582) | 100 | -0.087 | 0.347 (0.659) |
| Macaca_mulatta | 3 | 180294 | 53 | 6.196 | 5.002 | 0.053 | etienne | 0.32 (0.582) | 100 | -0.119 | 0.309 (0.659) |
| Macaca_mulatta | 2 | 190049 | 51 | 5.857 | 4.765 | 0.056 | etienne | 0.26 (0.582) | 100 | -0.027 | 0.427 (0.659) |
| Macaca_mulatta | 5 | 181430 | 51 | 5.844 | 4.789 | 0.061 | etienne | 0.38 (0.582) | 100 | -0.033 | 0.415 (0.659) |
| Macaca_mulatta | 4 | 164706 | 52 | 6.131 | 4.944 | 0.053 | etienne | 0.35 (0.582) | 100 | -0.071 | 0.366 (0.659) |
| Macaca_mulatta | 7 | 169871 | 50 | 5.971 | 4.716 | 0.042 | etienne | 0.33 (0.582) | 100 | -0.065 | 0.37 (0.659) |
| Macaca_mulatta | 6 | 174130 | 52 | 6.147 | 4.915 | 0.048 | etienne | 0.3 (0.582) | 100 | -0.074 | 0.366 (0.659) |
| Macaca_mulatta | 9 | 129385 | 51 | 5.945 | 4.965 | 0.082 | etienne | 0.26 (0.582) | 100 | -0.08 | 0.357 (0.659) |
| Macaca_mulatta | X | 145731 | 52 | 6.126 | 5.009 | 0.064 | etienne | 0.18 (0.582) | 100 | -0.1 | 0.325 (0.659) |
| Macaca_mulatta | 8 | 142045 | 51 | 6.133 | 4.915 | 0.052 | etienne | 0.28 (0.582) | 100 | -0.068 | 0.37 (0.659) |
| Gallus_gallus | 24 | 1823 | 24 | 3.805 | 3.803 | --- | ewens | 0.1 (0.582) | 100 | -0.353 | 0.141 (0.659) |
| Gallus_gallus | 25 | 591 | 14 | 2.489 | 2.454 | --- | ewens | 0.26 (0.582) | 100 | -0.005 | 0.459 (0.659) |
| Gallus_gallus | 26 | 1385 | 17 | 2.629 | 2.627 | --- | ewens | 0.09 (0.582) | 100 | -0.165 | 0.297 (0.659) |
| Gallus_gallus | 27 | 1831 | 19 | 2.954 | 2.858 | --- | ewens | 0.19 (0.582) | 100 | -0.075 | 0.384 (0.659) |
| Gallus_gallus | 20 | 3913 | 27 | 3.821 | 3.819 | --- | ewens | 0.11 (0.582) | 100 | -0.386 | 0.125 (0.659) |
| Gallus_gallus | 21 | 2142 | 19 | 2.78 | 2.778 | --- | ewens | 0.33 (0.582) | 100 | -0.19 | 0.275 (0.659) |
| Gallus_gallus | 22 | 1260 | 20 | 3.273 | 3.27 | --- | ewens | 0.07 (0.582) | 100 | -0.331 | 0.167 (0.659) |
| Gallus_gallus | 23 | 1772 | 23 | 3.632 | 3.63 | --- | ewens | 0.09 (0.582) | 100 | -0.382 | 0.128 (0.659) |
| Gallus_gallus | 28 | 1285 | 25 | 4.296 | 4.291 | --- | ewens | 0.17 (0.582) | 100 | -0.404 | 0.104 (0.659) |
| Gallus_gallus | 1 | 97336 | 34 | 3.644 | 3.247 | --- | ewens | 0.2 (0.582) | 100 | -0.37 | 0.148 (0.659) |
| Gallus_gallus | 3 | 46492 | 33 | 3.718 | 3.411 | --- | ewens | 0.12 (0.582) | 100 | -0.437 | 0.107 (0.659) |
| Gallus_gallus | 2 | 69279 | 35 | 3.72 | 3.483 | --- | ewens | 0.15 (0.582) | 100 | -0.449 | 0.1 (0.659) |
| Gallus_gallus | 5 | 21539 | 33 | 3.828 | 3.752 | --- | ewens | 0.09 (0.582) | 100 | -0.451 | 0.098 (0.659) |
| Gallus_gallus | 4 | 36276 | 34 | 3.674 | 3.636 | --- | ewens | 0.05 (0.582) | 100 | -0.471 | 0.091 (0.659) |
| Gallus_gallus | 7 | 11410 | 29 | 3.73 | 3.523 | --- | ewens | 0.15 (0.582) | 100 | -0.411 | 0.117 (0.659) |
| Gallus_gallus | 6 | 11101 | 30 | 3.82 | 3.679 | --- | ewens | 0.14 (0.582) | 100 | -0.445 | 0.102 (0.659) |
| Gallus_gallus | 9 | 6621 | 32 | 4.288 | 4.287 | --- | ewens | 0.06 (0.582) | 100 | -0.556 | 0.053 (0.659) |
| Gallus_gallus | 8 | 9733 | 30 | 3.796 | 3.749 | --- | ewens | 0.12 (0.582) | 100 | -0.441 | 0.104 (0.659) |
| Gallus_gallus | W | 167 | 10 | 2.202 | 2.175 | --- | ewens | 0.4 (0.582) | 100 | -0.034 | 0.425 (0.659) |
| Gallus_gallus | Z | 36254 | 31 | 3.3 | 3.272 | --- | ewens | 0.07 (0.582) | 100 | -0.368 | 0.146 (0.659) |
| Gallus_gallus | 11 | 6352 | 28 | 3.788 | 3.687 | --- | ewens | 0.05 (0.582) | 100 | -0.412 | 0.111 (0.659) |
| Gallus_gallus | 10 | 5972 | 27 | 3.618 | 3.566 | --- | ewens | 0.06 (0.582) | 100 | -0.385 | 0.13 (0.659) |
| Gallus_gallus | 13 | 4987 | 26 | 3.51 | 3.509 | --- | ewens | 0.12 (0.582) | 100 | -0.357 | 0.147 (0.659) |
| Gallus_gallus | 12 | 5407 | 28 | 3.783 | 3.782 | --- | ewens | 0.06 (0.582) | 100 | -0.439 | 0.1 (0.659) |
| Gallus_gallus | 15 | 3480 | 29 | 4.246 | 4.244 | --- | ewens | 0.02 (0.582) | 100 | -0.497 | 0.076 (0.659) |
| Gallus_gallus | 14 | 4225 | 24 | 3.279 | 3.278 | --- | ewens | 0.2 (0.582) | 100 | -0.275 | 0.204 (0.659) |
| Gallus_gallus | 17 | 3136 | 29 | 4.333 | 4.323 | --- | ewens | 0.07 (0.582) | 100 | -0.518 | 0.067 (0.659) |
| Gallus_gallus | 16 | 309 | 12 | 6.405 | 2.346 | --- | ewens | 0.72 (0.728) | 100 | 0.47 | 0.909 (0.917) |
| Gallus_gallus | 19 | 2733 | 22 | 3.2 | 3.178 | --- | ewens | 0.15 (0.582) | 100 | -0.251 | 0.225 (0.659) |
| Gallus_gallus | 18 | 3043 | 26 | 3.814 | 3.812 | --- | ewens | 0.05 (0.582) | 100 | -0.355 | 0.143 (0.659) |
| Rattus_norvegicus | 11 | 90560 | 52 | 6.039 | 5.281 | 0.151 | etienne | 0.3 (0.582) | 100 | -0.142 | 0.282 (0.659) |
| Rattus_norvegicus | 10 | 141217 | 53 | 5.918 | 5.134 | --- | ewens | 0.33 (0.582) | 100 | 0.113 | 0.615 (0.736) |
| Rattus_norvegicus | 13 | 118885 | 52 | 5.812 | 5.121 | 0.156 | etienne | 0.31 (0.582) | 100 | -0.093 | 0.34 (0.659) |
| Rattus_norvegicus | 12 | 66737 | 49 | 5.65 | 5.116 | --- | ewens | 0.39 (0.582) | 100 | 0.085 | 0.585 (0.717) |
| Rattus_norvegicus | 20 | 62561 | 49 | 5.7 | 5.156 | --- | ewens | 0.26 (0.582) | 100 | 0.048 | 0.53 (0.689) |
| Rattus_norvegicus | 14 | 111916 | 51 | 5.778 | 5.045 | --- | ewens | 0.36 (0.582) | 100 | 0.048 | 0.529 (0.688) |
| Rattus_norvegicus | 17 | 100531 | 53 | 5.897 | 5.331 | --- | ewens | 0.37 (0.582) | 100 | -0.003 | 0.462 (0.66) |
| Rattus_norvegicus | 16 | 89367 | 50 | 5.744 | 5.06 | --- | ewens | 0.26 (0.582) | 100 | 0.007 | 0.468 (0.664) |
| Rattus_norvegicus | 19 | 67686 | 50 | 5.926 | 5.226 | 0.176 | etienne | 0.35 (0.582) | 100 | -0.061 | 0.383 (0.659) |
| Rattus_norvegicus | 18 | 89202 | 50 | 5.926 | 5.061 | 0.115 | etienne | 0.37 (0.582) | 100 | -0.104 | 0.332 (0.659) |
| Rattus_norvegicus | 15 | 111442 | 53 | 5.832 | 5.27 | --- | ewens | 0.35 (0.582) | 100 | -0.011 | 0.446 (0.659) |
| Rattus_norvegicus | 1 | 281327 | 53 | 5.808 | 4.778 | 0.057 | etienne | 0.34 (0.582) | 100 | -0.021 | 0.435 (0.659) |
| Rattus_norvegicus | 3 | 182070 | 52 | 5.914 | 4.892 | 0.068 | etienne | 0.29 (0.582) | 100 | -0.042 | 0.404 (0.659) |
| Rattus_norvegicus | 2 | 250783 | 53 | 5.918 | 4.834 | 0.055 | etienne | 0.36 (0.582) | 100 | -0.136 | 0.289 (0.659) |
| Rattus_norvegicus | 5 | 186488 | 53 | 5.915 | 4.984 | 0.084 | etienne | 0.37 (0.582) | 100 | -0.028 | 0.423 (0.659) |
| Rattus_norvegicus | 4 | 193735 | 52 | 5.844 | 4.86 | 0.071 | etienne | 0.35 (0.582) | 100 | -0.076 | 0.358 (0.659) |
| Rattus_norvegicus | 7 | 152606 | 52 | 5.773 | 4.984 | 0.116 | etienne | 0.38 (0.582) | 100 | -0.03 | 0.42 (0.659) |
| Rattus_norvegicus | 6 | 150537 | 52 | 5.917 | 4.991 | 0.089 | etienne | 0.38 (0.582) | 100 | -0.079 | 0.358 (0.659) |
| Rattus_norvegicus | 9 | 119855 | 52 | 6.049 | 5.117 | 0.097 | etienne | 0.2 (0.582) | 100 | -0.13 | 0.299 (0.659) |
| Rattus_norvegicus | X | 157980 | 52 | 5.852 | 4.966 | 0.094 | etienne | 0.38 (0.582) | 100 | -0.082 | 0.357 (0.659) |
| Rattus_norvegicus | 8 | 144082 | 53 | 5.944 | 5.123 | 0.116 | etienne | 0.35 (0.582) | 100 | -0.043 | 0.4 (0.659) |
| Dictyostelium_discoideum | 1 | 26650 | 14 | 1.36 | 1.36 | --- | ewens | 0.1 (0.582) | 100 | -0.242 | 0.27 (0.659) |
| Dictyostelium_discoideum | 3 | 36422 | 12 | 1.103 | 1.098 | --- | ewens | 0.23 (0.582) | 100 | -0.109 | 0.382 (0.659) |
| Dictyostelium_discoideum | 2 | 47601 | 11 | 0.985 | 0.964 | --- | ewens | 0.28 (0.582) | 100 | -0.033 | 0.447 (0.659) |
| Dictyostelium_discoideum | 5 | 28146 | 12 | 1.129 | 1.129 | --- | ewens | 0.18 (0.582) | 100 | -0.146 | 0.349 (0.659) |
| Dictyostelium_discoideum | 4 | 31276 | 13 | 1.226 | 1.226 | --- | ewens | 0.15 (0.582) | 100 | -0.207 | 0.301 (0.659) |
| Dictyostelium_discoideum | 6 | 19250 | 9 | 0.894 | 0.837 | --- | ewens | 0.4 (0.582) | 100 | 0.09 | 0.563 (0.713) |
| Anopheles_gambiae | 2L | 27127 | 41 | 6.743 | 4.671 | 0.027 | etienne | 0.38 (0.582) | 100 | -0.516 | 0.042 (0.659) |
| Anopheles_gambiae | X | 21215 | 42 | 6.972 | 4.962 | 0.037 | etienne | 0.39 (0.582) | 100 | -0.582 | 0.029 (0.659) |
| Anopheles_gambiae | 3R | 26632 | 43 | 6.815 | 4.945 | 0.038 | etienne | 0.39 (0.582) | 100 | -0.537 | 0.036 (0.659) |
| Anopheles_gambiae | 2R | 31951 | 40 | 6.249 | 4.446 | 0.03 | etienne | 0.35 (0.582) | 100 | -0.596 | 0.032 (0.659) |
| Anopheles_gambiae | 3L | 21606 | 41 | 6.805 | 4.814 | 0.035 | etienne | 0.31 (0.582) | 100 | -0.445 | 0.065 (0.659) |
| Populus_trichocarpa | 11 | 22678 | 15 | 2.407 | 1.501 | --- | ewens | 0.53 (0.601) | 100 | 0.1 | 0.563 (0.713) |
| Populus_trichocarpa | 10 | 27535 | 15 | 2.291 | 1.468 | 0.01 | etienne | 0.54 (0.601) | 100 | -0.316 | 0.194 (0.659) |
| Populus_trichocarpa | 13 | 19984 | 15 | 2.422 | 1.524 | 0.01 | etienne | 0.46 (0.582) | 100 | -0.276 | 0.212 (0.659) |
| Populus_trichocarpa | 12 | 19675 | 15 | 2.4 | 1.527 | 0.011 | etienne | 0.46 (0.582) | 100 | -0.312 | 0.201 (0.659) |
| Populus_trichocarpa | 15 | 19796 | 15 | 2.406 | 1.525 | 0.011 | etienne | 0.53 (0.601) | 100 | -0.262 | 0.228 (0.659) |
| Populus_trichocarpa | 14 | 22978 | 15 | 2.188 | 1.499 | 0.017 | etienne | 0.65 (0.662) | 100 | -0.284 | 0.221 (0.659) |
| Populus_trichocarpa | 17 | 17858 | 15 | 2.287 | 1.544 | 0.017 | etienne | 0.6 (0.627) | 100 | -0.215 | 0.267 (0.659) |
| Populus_trichocarpa | 16 | 18927 | 15 | 2.379 | 1.534 | 0.012 | etienne | 0.46 (0.582) | 100 | -0.284 | 0.217 (0.659) |
| Populus_trichocarpa | 19 | 20777 | 15 | 2.43 | 1.517 | --- | ewens | 0.51 (0.593) | 100 | 0.067 | 0.533 (0.69) |
| Populus_trichocarpa | 18 | 19519 | 15 | 2.323 | 1.528 | --- | ewens | 0.53 (0.601) | 100 | -0.002 | 0.464 (0.661) |
| Populus_trichocarpa | 1 | 62281 | 15 | 2.368 | 1.344 | 0.003 | etienne | 0.54 (0.601) | 100 | -0.272 | 0.223 (0.659) |
| Populus_trichocarpa | 3 | 27266 | 15 | 2.211 | 1.47 | 0.013 | etienne | 0.45 (0.582) | 100 | -0.336 | 0.184 (0.659) |
| Populus_trichocarpa | 2 | 32946 | 15 | 2.232 | 1.439 | 0.009 | etienne | 0.47 (0.584) | 100 | -0.377 | 0.162 (0.659) |
| Populus_trichocarpa | 5 | 33143 | 15 | 2.317 | 1.438 | 0.007 | etienne | 0.44 (0.582) | 100 | -0.325 | 0.19 (0.659) |
| Populus_trichocarpa | 4 | 28944 | 15 | 2.328 | 1.46 | 0.008 | etienne | 0.55 (0.604) | 100 | -0.25 | 0.24 (0.659) |
| Populus_trichocarpa | 7 | 19958 | 15 | 2.382 | 1.524 | 0.011 | etienne | 0.57 (0.613) | 100 | -0.3 | 0.202 (0.659) |
| Populus_trichocarpa | 6 | 36091 | 15 | 2.267 | 1.424 | 0.008 | etienne | 0.62 (0.639) | 100 | -0.318 | 0.196 (0.659) |
| Populus_trichocarpa | 9 | 17324 | 15 | 2.221 | 1.55 | --- | ewens | 0.52 (0.599) | 100 | -0.111 | 0.368 (0.659) |
| Populus_trichocarpa | 8 | 23600 | 15 | 2.201 | 1.494 | 0.016 | etienne | 0.43 (0.582) | 100 | -0.339 | 0.184 (0.659) |
| Pongo_abelii | 20 | 66582 | 50 | 6.201 | 5.236 | 0.11 | etienne | 0.33 (0.582) | 100 | -0.075 | 0.362 (0.659) |
| Pongo_abelii | 21 | 34004 | 49 | 5.998 | 5.561 | --- | ewens | 0.11 (0.582) | 100 | -0.014 | 0.444 (0.659) |
| Pongo_abelii | 22 | 36554 | 48 | 6.091 | 5.381 | 0.202 | etienne | 0.14 (0.582) | 100 | -0.187 | 0.234 (0.659) |
| Pongo_abelii | 1 | 237604 | 51 | 6.155 | 4.657 | 0.025 | etienne | 0.28 (0.582) | 100 | -0.074 | 0.368 (0.659) |
| Pongo_abelii | 3 | 197243 | 52 | 6.161 | 4.851 | 0.04 | etienne | 0.21 (0.582) | 100 | -0.059 | 0.386 (0.659) |
| Pongo_abelii | 5 | 173705 | 52 | 6.23 | 4.916 | 0.042 | etienne | 0.27 (0.582) | 100 | -0.073 | 0.359 (0.659) |
| Pongo_abelii | 4 | 189323 | 53 | 6.133 | 4.976 | 0.055 | etienne | 0.24 (0.582) | 100 | -0.07 | 0.368 (0.659) |
| Pongo_abelii | 7 | 152312 | 52 | 6.287 | 4.985 | 0.046 | etienne | 0.27 (0.582) | 100 | -0.127 | 0.29 (0.659) |
| Pongo_abelii | 6 | 165756 | 52 | 6.298 | 4.94 | 0.04 | etienne | 0.3 (0.582) | 100 | -0.072 | 0.36 (0.659) |
| Pongo_abelii | 9 | 116601 | 50 | 6.035 | 4.912 | 0.064 | etienne | 0.28 (0.582) | 100 | -0.077 | 0.361 (0.659) |
| Pongo_abelii | 8 | 145474 | 51 | 6.189 | 4.902 | 0.046 | etienne | 0.41 (0.582) | 100 | -0.063 | 0.38 (0.659) |
| Pongo_abelii | X | 151577 | 52 | 6.224 | 4.988 | 0.051 | etienne | 0.21 (0.582) | 100 | -0.116 | 0.308 (0.659) |
| Pongo_abelii | 11 | 132034 | 51 | 6.186 | 4.954 | 0.053 | etienne | 0.28 (0.582) | 100 | -0.064 | 0.37 (0.659) |
| Pongo_abelii | 10 | 129018 | 51 | 6.059 | 4.966 | 0.068 | etienne | 0.39 (0.582) | 100 | -0.086 | 0.347 (0.659) |
| Pongo_abelii | 13 | 95671 | 52 | 6.271 | 5.248 | 0.092 | etienne | 0.28 (0.582) | 100 | -0.113 | 0.308 (0.659) |
| Pongo_abelii | 12 | 141041 | 50 | 6.164 | 4.811 | 0.04 | etienne | 0.33 (0.582) | 100 | -0.073 | 0.365 (0.659) |
| Pongo_abelii | 15 | 80921 | 51 | 6.216 | 5.234 | 0.102 | etienne | 0.19 (0.582) | 100 | -0.121 | 0.303 (0.659) |
| Pongo_abelii | 14 | 90285 | 50 | 6.202 | 5.054 | 0.07 | etienne | 0.32 (0.582) | 100 | -0.071 | 0.366 (0.659) |
| Pongo_abelii | 17 | 79055 | 49 | 6.14 | 5.016 | 0.074 | etienne | 0.23 (0.582) | 100 | -0.185 | 0.235 (0.659) |
| Pongo_abelii | 16 | 84930 | 51 | 6.144 | 5.204 | --- | ewens | 0.23 (0.582) | 100 | -0.015 | 0.44 (0.659) |
| Pongo_abelii | 19 | 69102 | 49 | 5.846 | 5.095 | 0.152 | etienne | 0.2 (0.582) | 100 | -0.277 | 0.158 (0.659) |
| Pongo_abelii | 18 | 72969 | 51 | 6.201 | 5.297 | 0.122 | etienne | 0.24 (0.582) | 100 | -0.094 | 0.339 (0.659) |
| Pongo_abelii | 2a | 107181 | 51 | 6.155 | 5.069 | 0.075 | etienne | 0.28 (0.582) | 100 | -0.074 | 0.362 (0.659) |
| Pongo_abelii | 2b | 125680 | 51 | 6.242 | 4.981 | 0.052 | etienne | 0.37 (0.582) | 100 | -0.07 | 0.368 (0.659) |
| Brachypodium_distachyon | 1 | 23837 | 9 | 3.283 | 0.818 | --- | ewens | 0.63 (0.648) | 100 | 0.664 | 0.946 (0.953) |
| Brachypodium_distachyon | 3 | 19442 | 9 | 3.649 | 0.837 | --- | ewens | 0.55 (0.604) | 100 | 0.67 | 0.953 (0.953) |
| Brachypodium_distachyon | 2 | 19277 | 9 | 3.798 | 0.837 | --- | ewens | 0.55 (0.604) | 100 | 0.67 | 0.953 (0.953) |
| Brachypodium_distachyon | 5 | 9329 | 9 | 3.358 | 0.911 | --- | ewens | 0.62 (0.639) | 100 | 0.666 | 0.952 (0.953) |
| Brachypodium_distachyon | 4 | 15474 | 9 | 3.473 | 0.858 | --- | ewens | 0.49 (0.587) | 100 | 0.679 | 0.952 (0.953) |
| Ciona_intestinalis | 1q | 7018 | 25 | 3.515 | 3.178 | --- | ewens | 0.34 (0.582) | 100 | 0.028 | 0.495 (0.674) |
| Ciona_intestinalis | 1p | 6143 | 24 | 3.203 | 3.091 | --- | ewens | 0.54 (0.601) | 100 | 0.091 | 0.569 (0.713) |
| Ciona_intestinalis | 10q | 4161 | 21 | 3.273 | 2.803 | --- | ewens | 0.48 (0.586) | 100 | 0.067 | 0.528 (0.688) |
| Ciona_intestinalis | 2q | 6380 | 27 | 3.531 | 3.53 | --- | ewens | 0.3 (0.582) | 100 | -0.105 | 0.351 (0.659) |
| Ciona_intestinalis | 14p | 3673 | 21 | 3.407 | 2.86 | --- | ewens | 0.38 (0.582) | 100 | 0.102 | 0.575 (0.714) |
| Ciona_intestinalis | 14q | 3121 | 20 | 3.113 | 2.771 | --- | ewens | 0.57 (0.613) | 100 | 0.253 | 0.739 (0.812) |
| Ciona_intestinalis | 3q | 4812 | 23 | 3.472 | 3.052 | --- | ewens | 0.35 (0.582) | 100 | 0.016 | 0.483 (0.671) |
| Ciona_intestinalis | 3p | 4127 | 24 | 3.651 | 3.29 | --- | ewens | 0.41 (0.582) | 100 | 0.122 | 0.609 (0.735) |
| Ciona_intestinalis | 5q | 5476 | 23 | 3.162 | 2.991 | --- | ewens | 0.39 (0.582) | 100 | -0.056 | 0.397 (0.659) |
| Ciona_intestinalis | 6q | 2556 | 22 | 3.264 | 3.215 | --- | ewens | 0.47 (0.584) | 100 | 0.105 | 0.586 (0.717) |
| Ciona_intestinalis | 4q | 5444 | 24 | 3.149 | 3.148 | --- | ewens | 0.29 (0.582) | 100 | -0.045 | 0.413 (0.659) |
| Ciona_intestinalis | 12p | 2101 | 20 | 2.968 | 2.967 | --- | ewens | 0.56 (0.607) | 100 | 0.102 | 0.581 (0.717) |
| Ciona_intestinalis | 12q | 4041 | 23 | 3.139 | 3.138 | --- | ewens | 0.46 (0.582) | 100 | 0.028 | 0.49 (0.672) |
| Ciona_intestinalis | 13q | 3290 | 19 | 2.584 | 2.583 | --- | ewens | 0.52 (0.599) | 100 | 0.211 | 0.694 (0.797) |
| Ciona_intestinalis | 13p | 553 | 15 | 9.296 | 2.721 | --- | ewens | 0.65 (0.662) | 100 | 0.279 | 0.773 (0.836) |
| Ciona_intestinalis | 7q | 7204 | 22 | 3.119 | 2.724 | --- | ewens | 0.46 (0.582) | 100 | 0.111 | 0.587 (0.717) |
| Ciona_intestinalis | 9q | 3825 | 24 | 3.332 | 3.331 | --- | ewens | 0.23 (0.582) | 100 | -0.081 | 0.369 (0.659) |
| Ciona_intestinalis | 9p | 3444 | 20 | 20 | 2.726 | --- | ewens | 0.44 (0.582) | 100 | 0.209 | 0.693 (0.797) |
| Ciona_intestinalis | 10p | 1362 | 17 | 3.732 | 2.635 | --- | ewens | 0.59 (0.623) | 100 | 0.274 | 0.767 (0.834) |
| Ciona_intestinalis | 8q | 6609 | 23 | 3.254 | 2.907 | --- | ewens | 0.36 (0.582) | 100 | 0.112 | 0.593 (0.722) |
| Bos_taurus | 24 | 66936 | 48 | 5.654 | 4.997 | 0.179 | etienne | 0.44 (0.582) | 100 | 0.114 | 0.625 (0.739) |
| Bos_taurus | 25 | 49840 | 46 | 5.435 | 4.933 | --- | ewens | 0.45 (0.582) | 100 | 0.308 | 0.854 (0.867) |
| Bos_taurus | 26 | 52611 | 48 | 5.741 | 5.143 | --- | ewens | 0.38 (0.582) | 100 | 0.255 | 0.805 (0.839) |
| Bos_taurus | 27 | 49156 | 48 | 5.708 | 5.186 | --- | ewens | 0.3 (0.582) | 100 | 0.197 | 0.732 (0.808) |
| Bos_taurus | 20 | 82304 | 50 | 5.108 | 5.108 | --- | ewens | 0.53 (0.601) | 100 | 0.213 | 0.749 (0.818) |
| Bos_taurus | 21 | 74680 | 48 | 5.781 | 4.933 | --- | ewens | 0.3 (0.582) | 100 | 0.288 | 0.833 (0.854) |
| Bos_taurus | 22 | 62032 | 48 | 5.298 | 5.042 | --- | ewens | 0.44 (0.582) | 100 | 0.307 | 0.851 (0.866) |
| Bos_taurus | 23 | 55004 | 46 | 5.556 | 4.874 | 0.17 | etienne | 0.59 (0.623) | 100 | 0.22 | 0.766 (0.834) |
| Bos_taurus | 28 | 47610 | 48 | 5.271 | 5.206 | --- | ewens | 0.48 (0.586) | 100 | 0.252 | 0.801 (0.839) |
| Bos_taurus | 29 | 54671 | 47 | 5.548 | 4.998 | --- | ewens | 0.45 (0.582) | 100 | 0.277 | 0.824 (0.848) |
| Bos_taurus | 1 | 173733 | 51 | 5.619 | 4.81 | 0.101 | etienne | 0.53 (0.601) | 100 | 0.156 | 0.682 (0.792) |
| Bos_taurus | 3 | 137438 | 51 | 5.767 | 4.932 | 0.106 | etienne | 0.35 (0.582) | 100 | 0.183 | 0.724 (0.805) |
| Bos_taurus | 2 | 151700 | 52 | 5.581 | 4.987 | --- | ewens | 0.45 (0.582) | 100 | 0.285 | 0.834 (0.854) |
| Bos_taurus | 5 | 136436 | 50 | 5.751 | 4.828 | 0.085 | etienne | 0.35 (0.582) | 100 | 0.174 | 0.706 (0.798) |
| Bos_taurus | 4 | 129996 | 51 | 5.619 | 4.962 | --- | ewens | 0.5 (0.59) | 100 | 0.258 | 0.799 (0.839) |
| Bos_taurus | 7 | 119063 | 52 | 5.588 | 5.121 | --- | ewens | 0.51 (0.593) | 100 | 0.27 | 0.813 (0.839) |
| Bos_taurus | 6 | 133800 | 52 | 5.54 | 5.056 | --- | ewens | 0.49 (0.587) | 100 | 0.233 | 0.776 (0.836) |
| Bos_taurus | 9 | 114389 | 50 | 5.624 | 4.922 | 0.144 | etienne | 0.28 (0.582) | 100 | 0.121 | 0.638 (0.749) |
| Bos_taurus | 8 | 122311 | 50 | 5.541 | 4.886 | 0.155 | etienne | 0.41 (0.582) | 100 | 0.188 | 0.721 (0.804) |
| Bos_taurus | X | 96715 | 50 | 5.364 | 5.015 | --- | ewens | 0.44 (0.582) | 100 | 0.248 | 0.788 (0.839) |
| Bos_taurus | 11 | 116694 | 52 | 5.255 | 5.132 | --- | ewens | 0.54 (0.601) | 100 | 0.256 | 0.8 (0.839) |
| Bos_taurus | 10 | 111050 | 50 | 5.703 | 4.938 | --- | ewens | 0.52 (0.599) | 100 | 0.324 | 0.861 (0.872) |
| Bos_taurus | 13 | 89332 | 50 | 5.512 | 5.06 | --- | ewens | 0.47 (0.584) | 100 | 0.313 | 0.852 (0.866) |
| Bos_taurus | 12 | 92648 | 51 | 5.305 | 5.153 | --- | ewens | 0.5 (0.59) | 100 | 0.173 | 0.703 (0.798) |
| Bos_taurus | 15 | 92607 | 52 | 5.267 | 5.267 | --- | ewens | 0.5 (0.59) | 100 | 0.228 | 0.775 (0.836) |
| Bos_taurus | 14 | 89877 | 48 | 5.572 | 4.83 | 0.134 | etienne | 0.47 (0.584) | 100 | 0.161 | 0.697 (0.797) |
| Bos_taurus | 17 | 83706 | 51 | 5.613 | 5.213 | --- | ewens | 0.4 (0.582) | 100 | 0.25 | 0.796 (0.839) |
| Bos_taurus | 16 | 82579 | 48 | 5.587 | 4.876 | --- | ewens | 0.43 (0.582) | 100 | 0.309 | 0.847 (0.864) |
| Bos_taurus | 19 | 67668 | 47 | 5.593 | 4.873 | --- | ewens | 0.43 (0.582) | 100 | 0.349 | 0.888 (0.898) |
| Bos_taurus | 18 | 69043 | 50 | 5.214 | 5.214 | --- | ewens | 0.46 (0.582) | 100 | 0.287 | 0.839 (0.858) |
| Zea_mays | 10 | 64454 | 26 | 3.83 | 2.508 | 0.01 | etienne | 0.59 (0.623) | 100 | -0.051 | 0.4 (0.659) |
| Zea_mays | 1 | 128837 | 26 | 3.851 | 2.332 | 0.004 | etienne | 0.56 (0.607) | 100 | -0.033 | 0.424 (0.659) |
| Zea_mays | 3 | 98150 | 26 | 3.867 | 2.398 | 0.005 | etienne | 0.62 (0.639) | 100 | -0.067 | 0.383 (0.659) |
| Zea_mays | 2 | 102160 | 26 | 3.777 | 2.388 | 0.006 | etienne | 0.65 (0.662) | 100 | -0.031 | 0.422 (0.659) |
| Zea_mays | 5 | 91848 | 26 | 3.851 | 2.415 | 0.006 | etienne | 0.51 (0.593) | 100 | -0.041 | 0.416 (0.659) |
| Zea_mays | 4 | 104732 | 26 | 3.816 | 2.382 | 0.006 | etienne | 0.54 (0.601) | 100 | -0.057 | 0.392 (0.659) |
| Zea_mays | 7 | 75981 | 26 | 3.857 | 2.464 | 0.008 | etienne | 0.59 (0.623) | 100 | -0.084 | 0.365 (0.659) |
| Zea_mays | 6 | 73546 | 26 | 3.983 | 2.472 | 0.006 | etienne | 0.6 (0.627) | 100 | -0.063 | 0.387 (0.659) |
| Zea_mays | 9 | 67117 | 26 | 3.92 | 2.497 | 0.008 | etienne | 0.62 (0.639) | 100 | -0.052 | 0.404 (0.659) |
| Zea_mays | 8 | 75019 | 26 | 3.872 | 2.467 | 0.008 | etienne | 0.59 (0.623) | 100 | -0.053 | 0.395 (0.659) |
| Sorghum_bicolor | 10 | 29589 | 23 | 2.829 | 2.383 | --- | ewens | 0.54 (0.601) | 100 | 0.334 | 0.812 (0.839) |
| Sorghum_bicolor | 1 | 37626 | 23 | 2.864 | 2.317 | 0.067 | etienne | 0.61 (0.635) | 100 | 0.195 | 0.684 (0.793) |
| Sorghum_bicolor | 3 | 37731 | 23 | 2.779 | 2.316 | --- | ewens | 0.64 (0.655) | 100 | 0.357 | 0.827 (0.851) |
| Sorghum_bicolor | 2 | 37915 | 23 | 2.867 | 2.315 | 0.066 | etienne | 0.53 (0.601) | 100 | 0.201 | 0.687 (0.794) |
| Sorghum_bicolor | 5 | 29326 | 23 | 2.857 | 2.386 | --- | ewens | 0.49 (0.587) | 100 | 0.313 | 0.789 (0.839) |
| Sorghum_bicolor | 4 | 32155 | 23 | 2.867 | 2.36 | --- | ewens | 0.62 (0.639) | 100 | 0.321 | 0.801 (0.839) |
| Sorghum_bicolor | 7 | 28471 | 23 | 2.866 | 2.394 | --- | ewens | 0.52 (0.599) | 100 | 0.292 | 0.774 (0.836) |
| Sorghum_bicolor | 6 | 29459 | 23 | 2.786 | 2.384 | --- | ewens | 0.56 (0.607) | 100 | 0.32 | 0.796 (0.839) |
| Sorghum_bicolor | 9 | 27546 | 23 | 2.725 | 2.404 | --- | ewens | 0.54 (0.601) | 100 | 0.306 | 0.786 (0.839) |
| Sorghum_bicolor | 8 | 25338 | 22 | 2.844 | 2.307 | --- | ewens | 0.55 (0.604) | 100 | 0.332 | 0.808 (0.839) |
| Tribolium_castaneum | 10 | 6319 | 14 | 1.628 | 1.628 | --- | ewens | 0.17 (0.582) | 100 | -0.686 | 0.064 (0.659) |
| Tribolium_castaneum | 1 | 3315 | 13 | 1.636 | 1.635 | --- | ewens | 0.21 (0.582) | 100 | -0.682 | 0.06 (0.659) |
| Tribolium_castaneum | 3 | 19294 | 20 | 2.144 | 2.137 | --- | ewens | 0.03 (0.582) | 100 | -0.939 | 0.018 (0.659) |
| Tribolium_castaneum | 2 | 5054 | 12 | 1.397 | 1.396 | --- | ewens | 0.09 (0.582) | 100 | -0.758 | 0.049 (0.659) |
| Tribolium_castaneum | 5 | 7837 | 16 | 1.913 | 1.851 | --- | ewens | 0.05 (0.582) | 100 | -0.903 | 0.023 (0.659) |
| Tribolium_castaneum | 4 | 6655 | 18 | 2.328 | 2.176 | --- | ewens | 0.12 (0.582) | 100 | -0.937 | 0.015 (0.659) |
| Tribolium_castaneum | 7 | 7865 | 18 | 2.3 | 2.125 | --- | ewens | 0.16 (0.582) | 100 | -0.974 | 0.015 (0.659) |
| Tribolium_castaneum | 6 | 8445 | 16 | 1.832 | 1.832 | --- | ewens | 0.07 (0.582) | 100 | -0.812 | 0.033 (0.659) |
| Tribolium_castaneum | 9 | 7824 | 16 | 1.851 | 1.851 | --- | ewens | 0.07 (0.582) | 100 | -0.838 | 0.029 (0.659) |
| Tribolium_castaneum | 8 | 10144 | 17 | 1.92 | 1.92 | --- | ewens | 0.07 (0.582) | 100 | -0.901 | 0.023 (0.659) |
| Thalassiosira_pseudonana | 16a | 200 | 6 | 1.035 | 1.029 | --- | ewens | 0.25 (0.582) | 100 | -0.354 | 0.207 (0.659) |
| Thalassiosira_pseudonana | 16b | 82 | 7 | 1.681 | 1.644 | --- | ewens | 0.33 (0.582) | 100 | -0.189 | 0.302 (0.659) |
| Thalassiosira_pseudonana | 24 | 152 | 9 | 1.989 | 1.934 | --- | ewens | 0.35 (0.582) | 100 | -0.25 | 0.251 (0.659) |
| Thalassiosira_pseudonana | 20 | 363 | 12 | 2.402 | 2.254 | --- | ewens | 0.14 (0.582) | 100 | -0.478 | 0.115 (0.659) |
| Thalassiosira_pseudonana | 22 | 453 | 12 | 2.318 | 2.139 | --- | ewens | 0.17 (0.582) | 100 | -0.478 | 0.119 (0.659) |
| Thalassiosira_pseudonana | 23 | 190 | 7 | 1.296 | 1.288 | --- | ewens | 0.14 (0.582) | 100 | -0.471 | 0.14 (0.659) |
| Thalassiosira_pseudonana | 1 | 1381 | 11 | 1.566 | 1.536 | --- | ewens | 0.12 (0.582) | 100 | -0.687 | 0.061 (0.659) |
| Thalassiosira_pseudonana | 3 | 1147 | 11 | 1.589 | 1.587 | --- | ewens | 0.16 (0.582) | 100 | -0.729 | 0.05 (0.659) |
| Thalassiosira_pseudonana | 2 | 1233 | 11 | 1.612 | 1.567 | --- | ewens | 0.23 (0.582) | 100 | -0.671 | 0.068 (0.659) |
| Thalassiosira_pseudonana | 5 | 1030 | 12 | 1.857 | 1.804 | --- | ewens | 0.14 (0.582) | 100 | -0.808 | 0.037 (0.659) |
| Thalassiosira_pseudonana | 4 | 1063 | 11 | 1.611 | 1.609 | --- | ewens | 0.07 (0.582) | 100 | -0.772 | 0.043 (0.659) |
| Thalassiosira_pseudonana | 7 | 871 | 12 | 1.866 | 1.863 | --- | ewens | 0.15 (0.582) | 100 | -0.705 | 0.05 (0.659) |
| Thalassiosira_pseudonana | 6 | 952 | 12 | 1.971 | 1.831 | --- | ewens | 0.08 (0.582) | 100 | -0.66 | 0.064 (0.659) |
| Thalassiosira_pseudonana | 9 | 488 | 11 | 1.885 | 1.88 | --- | ewens | 0.09 (0.582) | 100 | -0.704 | 0.052 (0.659) |
| Thalassiosira_pseudonana | 8 | 611 | 9 | 1.586 | 1.386 | --- | ewens | 0.45 (0.582) | 100 | -0.399 | 0.171 (0.659) |
| Thalassiosira_pseudonana | 11a | 349 | 9 | 1.568 | 1.561 | --- | ewens | 0.1 (0.582) | 100 | -0.493 | 0.126 (0.659) |
| Thalassiosira_pseudonana | 11b | 48 | 5 | 1.605 | 1.196 | --- | ewens | 0.57 (0.613) | 100 | 0.082 | 0.548 (0.701) |
| Thalassiosira_pseudonana | 10 | 508 | 11 | 1.909 | 1.864 | --- | ewens | 0.07 (0.582) | 100 | -0.618 | 0.075 (0.659) |
| Thalassiosira_pseudonana | 13 | 459 | 10 | 1.69 | 1.685 | --- | ewens | 0.02 (0.582) | 100 | -0.698 | 0.055 (0.659) |
| Thalassiosira_pseudonana | 12 | 466 | 12 | 2.13 | 2.125 | --- | ewens | 0.1 (0.582) | 100 | -0.651 | 0.065 (0.659) |
| Thalassiosira_pseudonana | 15 | 397 | 12 | 2.214 | 2.206 | --- | ewens | 0.05 (0.582) | 100 | -0.687 | 0.051 (0.659) |
| Thalassiosira_pseudonana | 14 | 420 | 12 | 2.183 | 2.176 | --- | ewens | 0.02 (0.582) | 100 | -0.877 | 0.024 (0.659) |
| Thalassiosira_pseudonana | 17 | 287 | 11 | 2.148 | 2.131 | --- | ewens | 0.1 (0.582) | 100 | -0.646 | 0.061 (0.659) |
| Thalassiosira_pseudonana | 18 | 403 | 9 | 1.516 | 1.512 | --- | ewens | 0.12 (0.582) | 100 | -0.461 | 0.14 (0.659) |
| Equus_caballus | 24 | 39603 | 45 | 5.595 | 4.949 | --- | ewens | 0.37 (0.582) | 100 | 0.206 | 0.739 (0.812) |
| Equus_caballus | 25 | 36317 | 45 | 5.345 | 5.004 | --- | ewens | 0.26 (0.582) | 100 | 0.128 | 0.638 (0.749) |
| Equus_caballus | 26 | 34226 | 44 | 5.385 | 4.914 | --- | ewens | 0.51 (0.593) | 100 | 0.207 | 0.741 (0.812) |
| Equus_caballus | 27 | 32130 | 45 | 5.393 | 5.083 | --- | ewens | 0.33 (0.582) | 100 | 0.159 | 0.68 (0.791) |
| Equus_caballus | 20 | 52856 | 47 | 5.826 | 5.019 | 0.141 | etienne | 0.44 (0.582) | 100 | 0.086 | 0.583 (0.717) |
| Equus_caballus | 21 | 47392 | 47 | 5.524 | 5.086 | --- | ewens | 0.37 (0.582) | 100 | 0.192 | 0.722 (0.804) |
| Equus_caballus | 22 | 47119 | 46 | 5.091 | 4.967 | --- | ewens | 0.62 (0.639) | 100 | 0.194 | 0.718 (0.804) |
| Equus_caballus | 23 | 46748 | 47 | 5.711 | 5.094 | --- | ewens | 0.46 (0.582) | 100 | 0.183 | 0.714 (0.803) |
| Equus_caballus | 28 | 41125 | 46 | 5.699 | 5.051 | --- | ewens | 0.32 (0.582) | 100 | 0.17 | 0.695 (0.797) |
| Equus_caballus | 29 | 26555 | 46 | 5.665 | 5.344 | --- | ewens | 0.38 (0.582) | 100 | 0.109 | 0.615 (0.736) |
| Equus_caballus | 1 | 156042 | 49 | 5.676 | 4.653 | 0.063 | etienne | 0.35 (0.582) | 100 | 0.072 | 0.565 (0.713) |
| Equus_caballus | 3 | 106428 | 47 | 5.355 | 4.628 | 0.124 | etienne | 0.49 (0.587) | 100 | 0.116 | 0.626 (0.739) |
| Equus_caballus | 2 | 107597 | 48 | 5.523 | 4.733 | 0.113 | etienne | 0.35 (0.582) | 100 | 0.071 | 0.566 (0.713) |
| Equus_caballus | 5 | 84889 | 46 | 5.688 | 4.634 | 0.069 | etienne | 0.48 (0.586) | 100 | 0.084 | 0.583 (0.717) |
| Equus_caballus | 4 | 89121 | 48 | 5.698 | 4.834 | 0.106 | etienne | 0.37 (0.582) | 100 | 0.085 | 0.582 (0.717) |
| Equus_caballus | 7 | 86189 | 47 | 5.565 | 4.739 | --- | ewens | 0.35 (0.582) | 100 | 0.212 | 0.746 (0.816) |
| Equus_caballus | 6 | 71089 | 49 | 5.426 | 5.079 | --- | ewens | 0.45 (0.582) | 100 | 0.189 | 0.72 (0.804) |
| Equus_caballus | 9 | 73193 | 48 | 5.503 | 4.945 | --- | ewens | 0.32 (0.582) | 100 | 0.195 | 0.726 (0.805) |
| Equus_caballus | 8 | 79489 | 48 | 5.448 | 4.898 | --- | ewens | 0.44 (0.582) | 100 | 0.195 | 0.727 (0.805) |
| Equus_caballus | X | 105268 | 50 | 5.868 | 4.968 | 0.1 | etienne | 0.39 (0.582) | 100 | 0.024 | 0.495 (0.674) |
| Equus_caballus | 11 | 52972 | 47 | 5.339 | 5.017 | --- | ewens | 0.45 (0.582) | 100 | 0.174 | 0.703 (0.798) |
| Equus_caballus | 10 | 71665 | 48 | 5.652 | 4.957 | 0.162 | etienne | 0.35 (0.582) | 100 | 0.09 | 0.588 (0.717) |
| Equus_caballus | 13 | 38471 | 47 | 5.524 | 5.22 | --- | ewens | 0.35 (0.582) | 100 | 0.138 | 0.654 (0.766) |
| Equus_caballus | 12 | 27660 | 46 | 5.361 | 5.315 | --- | ewens | 0.44 (0.582) | 100 | 0.11 | 0.62 (0.737) |
| Equus_caballus | 15 | 77468 | 48 | 5.557 | 4.912 | 0.174 | etienne | 0.44 (0.582) | 100 | 0.09 | 0.594 (0.722) |
| Equus_caballus | 14 | 82183 | 49 | 5.653 | 4.994 | --- | ewens | 0.31 (0.582) | 100 | 0.182 | 0.707 (0.798) |
| Equus_caballus | 17 | 67717 | 47 | 5.463 | 4.873 | --- | ewens | 0.37 (0.582) | 100 | 0.189 | 0.715 (0.803) |
| Equus_caballus | 16 | 77135 | 46 | 5.432 | 4.684 | 0.13 | etienne | 0.39 (0.582) | 100 | 0.12 | 0.634 (0.748) |
| Equus_caballus | 19 | 51052 | 44 | 5.484 | 4.676 | 0.126 | etienne | 0.47 (0.584) | 100 | 0.109 | 0.617 (0.737) |
| Equus_caballus | 18 | 68131 | 45 | 5.491 | 4.636 | 0.106 | etienne | 0.49 (0.587) | 100 | 0.098 | 0.606 (0.733) |
| Equus_caballus | 31 | 20073 | 44 | 5.511 | 5.274 | --- | ewens | 0.36 (0.582) | 100 | 0.152 | 0.674 (0.786) |
| Equus_caballus | 30 | 25502 | 43 | 5.345 | 4.973 | --- | ewens | 0.39 (0.582) | 100 | 0.159 | 0.674 (0.786) |
| Taeniopygia_guttata | 1A | 26965 | 29 | 3.224 | 3.144 | --- | ewens | 0.11 (0.582) | 100 | -0.392 | 0.133 (0.659) |
| Taeniopygia_guttata | 1B | 682 | 10 | 10 | 1.552 | --- | ewens | 0.42 (0.582) | 100 | 0.249 | 0.708 (0.798) |
| Taeniopygia_guttata | 24 | 3882 | 23 | 3.16 | 3.159 | --- | ewens | 0.19 (0.582) | 100 | -0.147 | 0.305 (0.659) |
| Taeniopygia_guttata | 25 | 776 | 9 | 5.894 | 1.324 | --- | ewens | 0.55 (0.604) | 100 | 0.377 | 0.811 (0.839) |
| Taeniopygia_guttata | 26 | 2083 | 16 | 2.267 | 2.266 | --- | ewens | 0.35 (0.582) | 100 | 0.09 | 0.559 (0.713) |
| Taeniopygia_guttata | 27 | 2220 | 13 | 13 | 1.741 | --- | ewens | 0.36 (0.582) | 100 | 0.275 | 0.739 (0.812) |
| Taeniopygia_guttata | 20 | 5207 | 22 | 2.861 | 2.86 | --- | ewens | 0.34 (0.582) | 100 | -0.074 | 0.377 (0.659) |
| Taeniopygia_guttata | 21 | 2643 | 16 | 2.266 | 2.177 | --- | ewens | 0.41 (0.582) | 100 | 0.018 | 0.479 (0.671) |
| Taeniopygia_guttata | 22 | 1688 | 16 | 2.353 | 2.351 | --- | ewens | 0.4 (0.582) | 100 | 0.131 | 0.602 (0.729) |
| Taeniopygia_guttata | 23 | 2820 | 19 | 2.651 | 2.649 | --- | ewens | 0.24 (0.582) | 100 | 0.047 | 0.507 (0.682) |
| Taeniopygia_guttata | 28 | 2529 | 21 | 3.047 | 3.045 | --- | ewens | 0.16 (0.582) | 100 | -0.099 | 0.354 (0.659) |
| Taeniopygia_guttata | 1 | 47271 | 29 | 3.16 | 2.94 | --- | ewens | 0.15 (0.582) | 100 | -0.343 | 0.162 (0.659) |
| Taeniopygia_guttata | 3 | 40441 | 31 | 3.23 | 3.23 | --- | ewens | 0.1 (0.582) | 100 | -0.442 | 0.105 (0.659) |
| Taeniopygia_guttata | 2 | 59490 | 33 | 3.533 | 3.315 | --- | ewens | 0.07 (0.582) | 100 | -0.445 | 0.107 (0.659) |
| Taeniopygia_guttata | 5 | 22206 | 27 | 2.968 | 2.968 | --- | ewens | 0.16 (0.582) | 100 | -0.318 | 0.179 (0.659) |
| Taeniopygia_guttata | 4 | 24316 | 31 | 3.438 | 3.438 | --- | ewens | 0.05 (0.582) | 100 | -0.514 | 0.079 (0.659) |
| Taeniopygia_guttata | 7 | 13378 | 27 | 3.172 | 3.172 | --- | ewens | 0.14 (0.582) | 100 | -0.4 | 0.127 (0.659) |
| Taeniopygia_guttata | 6 | 11683 | 25 | 2.955 | 2.955 | --- | ewens | 0.17 (0.582) | 100 | -0.275 | 0.206 (0.659) |
| Taeniopygia_guttata | 9 | 8724 | 29 | 3.663 | 3.662 | --- | ewens | 0.08 (0.582) | 100 | -0.426 | 0.108 (0.659) |
| Taeniopygia_guttata | 8 | 9445 | 25 | 3.249 | 3.044 | --- | ewens | 0.18 (0.582) | 100 | -0.337 | 0.163 (0.659) |
| Taeniopygia_guttata | Z | 31775 | 30 | 3.203 | 3.203 | --- | ewens | 0.07 (0.582) | 100 | -0.365 | 0.153 (0.659) |
| Taeniopygia_guttata | 4A | 6657 | 23 | 2.905 | 2.904 | --- | ewens | 0.24 (0.582) | 100 | -0.193 | 0.275 (0.659) |
| Taeniopygia_guttata | 11 | 7304 | 23 | 2.923 | 2.865 | --- | ewens | 0.21 (0.582) | 100 | -0.237 | 0.241 (0.659) |
| Taeniopygia_guttata | 10 | 6149 | 26 | 3.397 | 3.396 | --- | ewens | 0.18 (0.582) | 100 | -0.395 | 0.128 (0.659) |
| Taeniopygia_guttata | 13 | 5538 | 23 | 2.987 | 2.986 | --- | ewens | 0.2 (0.582) | 100 | -0.255 | 0.222 (0.659) |
| Taeniopygia_guttata | 12 | 6772 | 26 | 3.347 | 3.346 | --- | ewens | 0.13 (0.582) | 100 | -0.322 | 0.166 (0.659) |
| Taeniopygia_guttata | 15 | 4599 | 22 | 2.917 | 2.916 | --- | ewens | 0.22 (0.582) | 100 | -0.134 | 0.324 (0.659) |
| Taeniopygia_guttata | 14 | 5264 | 23 | 3.011 | 3.009 | --- | ewens | 0.17 (0.582) | 100 | -0.192 | 0.273 (0.659) |
| Taeniopygia_guttata | 17 | 4134 | 25 | 3.454 | 3.453 | --- | ewens | 0.07 (0.582) | 100 | -0.216 | 0.244 (0.659) |
| Taeniopygia_guttata | 19 | 4070 | 22 | 2.974 | 2.973 | --- | ewens | 0.12 (0.582) | 100 | -0.12 | 0.338 (0.659) |
| Taeniopygia_guttata | 18 | 4196 | 25 | 3.445 | 3.445 | --- | ewens | 0.06 (0.582) | 100 | -0.239 | 0.224 (0.659) |
| Mus_musculus | 11 | 165425 | 55 | 6.218 | 5.261 | 0.091 | etienne | 0.27 (0.582) | 100 | -0.127 | 0.299 (0.659) |
| Mus_musculus | 10 | 158644 | 53 | 6 | 5.07 | 0.09 | etienne | 0.24 (0.582) | 100 | -0.18 | 0.24 (0.659) |
| Mus_musculus | 13 | 138449 | 57 | 6.387 | 5.582 | 0.14 | etienne | 0.18 (0.582) | 100 | -0.213 | 0.207 (0.659) |
| Mus_musculus | 12 | 142367 | 59 | 6.691 | 5.784 | 0.123 | etienne | 0.19 (0.582) | 100 | -0.245 | 0.176 (0.659) |
| Mus_musculus | 15 | 120926 | 55 | 6.239 | 5.444 | 0.14 | etienne | 0.18 (0.582) | 100 | -0.207 | 0.213 (0.659) |
| Mus_musculus | 14 | 143018 | 59 | 6.579 | 5.781 | 0.149 | etienne | 0.15 (0.582) | 100 | -0.276 | 0.147 (0.659) |
| Mus_musculus | 17 | 114480 | 54 | 6.184 | 5.366 | 0.132 | etienne | 0.17 (0.582) | 100 | -0.181 | 0.236 (0.659) |
| Mus_musculus | 16 | 116244 | 55 | 6.367 | 5.468 | 0.118 | etienne | 0.16 (0.582) | 100 | -0.244 | 0.178 (0.659) |
| Mus_musculus | 19 | 74403 | 53 | 6.356 | 5.519 | 0.146 | etienne | 0.11 (0.582) | 100 | -0.17 | 0.246 (0.659) |
| Mus_musculus | 18 | 105225 | 52 | 6.199 | 5.192 | 0.09 | etienne | 0.2 (0.582) | 100 | -0.215 | 0.207 (0.659) |
| Mus_musculus | 1 | 233535 | 54 | 6.167 | 4.972 | 0.049 | etienne | 0.25 (0.582) | 100 | -0.202 | 0.215 (0.659) |
| Mus_musculus | 9 | 156215 | 55 | 6.289 | 5.294 | 0.087 | etienne | 0.27 (0.582) | 100 | -0.162 | 0.26 (0.659) |
| Mus_musculus | 3 | 182236 | 54 | 6.179 | 5.102 | 0.067 | etienne | 0.26 (0.582) | 100 | -0.236 | 0.19 (0.659) |
| Mus_musculus | 2 | 220766 | 55 | 6.292 | 5.104 | 0.053 | etienne | 0.21 (0.582) | 100 | -0.168 | 0.251 (0.659) |
| Mus_musculus | 5 | 194281 | 54 | 6.081 | 5.068 | 0.073 | etienne | 0.24 (0.582) | 100 | -0.155 | 0.266 (0.659) |
| Mus_musculus | 4 | 193463 | 56 | 6.264 | 5.28 | 0.084 | etienne | 0.22 (0.582) | 100 | -0.154 | 0.265 (0.659) |
| Mus_musculus | 7 | 177149 | 53 | 5.954 | 5.011 | 0.084 | etienne | 0.23 (0.582) | 100 | -0.088 | 0.341 (0.659) |
| Mus_musculus | 6 | 178519 | 58 | 6.534 | 5.538 | 0.092 | etienne | 0.16 (0.582) | 100 | -0.238 | 0.184 (0.659) |
| Mus_musculus | Y | 3160 | 27 | 3.965 | 3.962 | --- | ewens | 0.42 (0.582) | 100 | 0.048 | 0.521 (0.688) |
| Mus_musculus | X | 192089 | 54 | 6.214 | 5.074 | 0.059 | etienne | 0.18 (0.582) | 100 | -0.219 | 0.205 (0.659) |
| Mus_musculus | 8 | 156738 | 55 | 6.307 | 5.292 | 0.084 | etienne | 0.27 (0.582) | 100 | -0.215 | 0.213 (0.659) |
| Danio_rerio | 24 | 79346 | 57 | 56.579 | 5.947 | --- | ewens | 0.44 (0.582) | 100 | 0.163 | 0.7 (0.798) |
| Danio_rerio | 25 | 67546 | 54 | 7.998 | 5.702 | 0.025 | etienne | 0.43 (0.582) | 100 | -0.083 | 0.344 (0.659) |
| Danio_rerio | 20 | 96018 | 58 | 8.363 | 5.931 | 0.021 | etienne | 0.5 (0.59) | 100 | -0.05 | 0.392 (0.659) |
| Danio_rerio | 21 | 78595 | 54 | 8.215 | 5.601 | 0.017 | etienne | 0.46 (0.582) | 100 | -0.058 | 0.375 (0.659) |
| Danio_rerio | 22 | 72143 | 56 | 9.202 | 5.895 | 0.012 | etienne | 0.55 (0.604) | 100 | -0.009 | 0.453 (0.659) |
| Danio_rerio | 23 | 80567 | 53 | 8.246 | 5.468 | 0.014 | etienne | 0.56 (0.607) | 100 | -0.041 | 0.403 (0.659) |
| Danio_rerio | 1 | 105305 | 56 | 8.179 | 5.642 | 0.016 | etienne | 0.41 (0.582) | 100 | -0.115 | 0.297 (0.659) |
| Danio_rerio | 3 | 104327 | 61 | 8.462 | 6.218 | 0.028 | etienne | 0.53 (0.601) | 100 | -0.027 | 0.42 (0.659) |
| Danio_rerio | 2 | 102643 | 56 | 8.372 | 5.658 | 0.014 | etienne | 0.59 (0.623) | 100 | -0.027 | 0.425 (0.659) |
| Danio_rerio | 5 | 130528 | 57 | 8.648 | 5.619 | 0.01 | etienne | 0.46 (0.582) | 100 | -0.078 | 0.343 (0.659) |
| Danio_rerio | 4 | 92404 | 58 | 11.067 | 5.958 | 0.004 | etienne | 0.56 (0.607) | 100 | -0.09 | 0.316 (0.659) |
| Danio_rerio | 7 | 128203 | 57 | 8.25 | 5.63 | 0.014 | etienne | 0.5 (0.59) | 100 | -0.051 | 0.388 (0.659) |
| Danio_rerio | 6 | 104535 | 56 | 8.008 | 5.647 | 0.019 | etienne | 0.48 (0.586) | 100 | -0.069 | 0.362 (0.659) |
| Danio_rerio | 9 | 100725 | 55 | 8.165 | 5.557 | 0.015 | etienne | 0.48 (0.586) | 100 | -0.058 | 0.373 (0.659) |
| Danio_rerio | 8 | 97168 | 57 | 8.59 | 5.808 | 0.015 | etienne | 0.59 (0.623) | 100 | -0.046 | 0.391 (0.659) |
| Danio_rerio | 11 | 84518 | 55 | 8.066 | 5.67 | 0.021 | etienne | 0.44 (0.582) | 100 | -0.083 | 0.338 (0.659) |
| Danio_rerio | 10 | 80530 | 55 | 8.34 | 5.702 | 0.017 | etienne | 0.45 (0.582) | 100 | -0.061 | 0.372 (0.659) |
| Danio_rerio | 13 | 94729 | 57 | 8.442 | 5.825 | 0.017 | etienne | 0.4 (0.582) | 100 | -0.104 | 0.314 (0.659) |
| Danio_rerio | 12 | 90169 | 55 | 7.987 | 5.628 | 0.02 | etienne | 0.5 (0.59) | 100 | -0.075 | 0.357 (0.659) |
| Danio_rerio | 15 | 82583 | 55 | 8.422 | 5.685 | 0.016 | etienne | 0.48 (0.586) | 100 | -0.093 | 0.325 (0.659) |
| Danio_rerio | 14 | 92287 | 54 | 7.791 | 5.498 | 0.02 | etienne | 0.6 (0.627) | 100 | -0.045 | 0.4 (0.659) |
| Danio_rerio | 17 | 93951 | 55 | 8.008 | 5.601 | 0.019 | etienne | 0.5 (0.59) | 100 | -0.041 | 0.404 (0.659) |
| Danio_rerio | 16 | 102284 | 55 | 7.962 | 5.547 | 0.018 | etienne | 0.57 (0.613) | 100 | -0.033 | 0.414 (0.659) |
| Danio_rerio | 19 | 89357 | 54 | 8.094 | 5.518 | 0.016 | etienne | 0.48 (0.586) | 100 | -0.075 | 0.358 (0.659) |
| Danio_rerio | 18 | 90695 | 57 | 7.908 | 5.855 | 0.031 | etienne | 0.42 (0.582) | 100 | -0.085 | 0.34 (0.659) |
| Saccharomyces_cerevisiae | IX | 350 | 8 | 1.379 | 1.336 | --- | ewens | 0.38 (0.582) | 100 | -0.164 | 0.326 (0.659) |
| Saccharomyces_cerevisiae | VI | 236 | 7 | 6.241 | 1.222 | --- | ewens | 0.49 (0.587) | 100 | 0.105 | 0.568 (0.713) |
| Saccharomyces_cerevisiae | XI | 532 | 6 | 1.107 | 0.838 | --- | ewens | 0.56 (0.607) | 100 | 0.162 | 0.622 (0.737) |
| Saccharomyces_cerevisiae | I | 188 | 8 | 1.561 | 1.551 | --- | ewens | 0.28 (0.582) | 100 | -0.176 | 0.319 (0.659) |
| Saccharomyces_cerevisiae | XII | 846 | 10 | 1.57 | 1.489 | --- | ewens | 0.38 (0.582) | 100 | -0.207 | 0.293 (0.659) |
| Saccharomyces_cerevisiae | VII | 886 | 9 | 9 | 1.292 | --- | ewens | 0.41 (0.582) | 100 | -0.055 | 0.419 (0.659) |
| Saccharomyces_cerevisiae | IV | 1182 | 8 | 1.063 | 1.058 | --- | ewens | 0.46 (0.582) | 100 | -0.094 | 0.393 (0.659) |
| Saccharomyces_cerevisiae | XVI | 762 | 8 | 1.391 | 1.143 | --- | ewens | 0.46 (0.582) | 100 | -0.02 | 0.455 (0.659) |
| Saccharomyces_cerevisiae | II | 640 | 9 | 1.376 | 1.374 | --- | ewens | 0.3 (0.582) | 100 | -0.253 | 0.261 (0.659) |
| Saccharomyces_cerevisiae | XIV | 610 | 8 | 1.285 | 1.191 | --- | ewens | 0.39 (0.582) | 100 | -0.162 | 0.335 (0.659) |
| Saccharomyces_cerevisiae | V | 486 | 9 | 1.457 | 1.452 | --- | ewens | 0.46 (0.582) | 100 | -0.128 | 0.351 (0.659) |
| Saccharomyces_cerevisiae | X | 574 | 7 | 7 | 1.013 | --- | ewens | 0.54 (0.601) | 100 | 0.014 | 0.487 (0.672) |
| Saccharomyces_cerevisiae | III | 292 | 7 | 1.579 | 1.164 | --- | ewens | 0.58 (0.62) | 100 | 0.082 | 0.543 (0.697) |
| Saccharomyces_cerevisiae | XV | 872 | 8 | 1.398 | 1.115 | --- | ewens | 0.42 (0.582) | 100 | -0.037 | 0.441 (0.659) |
| Tetraodon_nigroviridis | 11 | 6346 | 12 | 1.366 | 1.354 | --- | ewens | 0.26 (0.582) | 100 | -0.247 | 0.266 (0.659) |
| Tetraodon_nigroviridis | 10 | 6908 | 11 | 1.206 | 1.206 | --- | ewens | 0.21 (0.582) | 100 | -0.169 | 0.323 (0.659) |
| Tetraodon_nigroviridis | 13 | 6760 | 12 | 1.371 | 1.342 | --- | ewens | 0.27 (0.582) | 100 | -0.226 | 0.278 (0.659) |
| Tetraodon_nigroviridis | 12 | 6752 | 11 | 1.216 | 1.209 | --- | ewens | 0.28 (0.582) | 100 | -0.194 | 0.303 (0.659) |
| Tetraodon_nigroviridis | 20 | 1451 | 7 | 0.864 | 0.863 | --- | ewens | 0.35 (0.582) | 100 | 0.045 | 0.525 (0.688) |
| Tetraodon_nigroviridis | 21 | 3136 | 10 | 1.199 | 1.199 | --- | ewens | 0.22 (0.582) | 100 | -0.239 | 0.277 (0.659) |
| Tetraodon_nigroviridis | 17 | 6269 | 12 | 1.356 | 1.356 | --- | ewens | 0.19 (0.582) | 100 | -0.299 | 0.231 (0.659) |
| Tetraodon_nigroviridis | 16 | 4373 | 9 | 1.006 | 1.005 | --- | ewens | 0.29 (0.582) | 100 | -0.053 | 0.429 (0.659) |
| Tetraodon_nigroviridis | 19 | 3941 | 8 | 0.882 | 0.882 | --- | ewens | 0.35 (0.582) | 100 | -0.041 | 0.445 (0.659) |
| Tetraodon_nigroviridis | 18 | 6423 | 12 | 1.412 | 1.351 | --- | ewens | 0.16 (0.582) | 100 | -0.269 | 0.25 (0.659) |
| Tetraodon_nigroviridis | 1 | 12361 | 14 | 1.49 | 1.49 | --- | ewens | 0.18 (0.582) | 100 | -0.342 | 0.201 (0.659) |
| Tetraodon_nigroviridis | 3 | 8505 | 17 | 2.055 | 1.966 | --- | ewens | 0.1 (0.582) | 100 | -0.49 | 0.114 (0.659) |
| Tetraodon_nigroviridis | 2 | 11616 | 13 | 1.376 | 1.376 | --- | ewens | 0.28 (0.582) | 100 | -0.257 | 0.257 (0.659) |
| Tetraodon_nigroviridis | 5 | 7267 | 11 | 1.198 | 1.198 | --- | ewens | 0.24 (0.582) | 100 | -0.215 | 0.293 (0.659) |
| Tetraodon_nigroviridis | 4 | 5009 | 10 | 1.168 | 1.122 | --- | ewens | 0.31 (0.582) | 100 | -0.096 | 0.387 (0.659) |
| Tetraodon_nigroviridis | 7 | 5944 | 10 | 1.244 | 1.097 | --- | ewens | 0.4 (0.582) | 100 | -0.083 | 0.403 (0.659) |
| Tetraodon_nigroviridis | 6 | 3345 | 10 | 1.244 | 1.188 | --- | ewens | 0.22 (0.582) | 100 | -0.177 | 0.33 (0.659) |
| Tetraodon_nigroviridis | 9 | 5236 | 12 | 1.415 | 1.39 | --- | ewens | 0.25 (0.582) | 100 | -0.272 | 0.251 (0.659) |
| Tetraodon_nigroviridis | 15 | 3672 | 11 | 1.316 | 1.316 | --- | ewens | 0.12 (0.582) | 100 | -0.225 | 0.283 (0.659) |
| Tetraodon_nigroviridis | 14 | 5482 | 13 | 1.52 | 1.52 | --- | ewens | 0.12 (0.582) | 100 | -0.354 | 0.196 (0.659) |
| Tetraodon_nigroviridis | 8 | 5746 | 11 | 1.243 | 1.236 | --- | ewens | 0.27 (0.582) | 100 | -0.216 | 0.291 (0.659) |
